# Hyaluronic acid–GPRC5C signalling promotes dormancy in haematopoietic stem cells

**DOI:** 10.1038/s41556-022-00931-x

**Published:** 2022-06-20

**Authors:** Yu Wei Zhang, Julian Mess, Nadim Aizarani, Pankaj Mishra, Carys Johnson, Mari Carmen Romero-Mulero, Jasmin Rettkowski, Katharina Schönberger, Nadine Obier, Karin Jäcklein, Nadine M. Woessner, Maria-Eleni Lalioti, Talia Velasco-Hernandez, Katarzyna Sikora, Ralph Wäsch, Bernhard Lehnertz, Guy Sauvageau, Thomas Manke, Pablo Menendez, Sebastian Gottfried Walter, Susana Minguet, Elisa Laurenti, Stefan Günther, Dominic Grün, Nina Cabezas-Wallscheid

**Affiliations:** 1 Max Planck Institute of Immunobiology and Epigenetics, Freiburg, Germany; 2 Faculty of Biology, University of Freiburg, Freiburg, Germany; 3 International Max Planck Research School for Molecular and Cellular Biology (IMPRS-MCB), Freiburg, Germany; 4 Spemann Graduate School for Biology and Medicine (SGBM), Freiburg, Germany; 5 Centre for Integrative Biological Signalling Studies (CIBSS), Freiburg, Germany; 6 Pharmaceutical Bioinformatics, University of Freiburg, Freiburg, Germany; 7 Department of Haematology and Wellcome and MRC Cambridge Stem Cell Institute, University of Cambridge, Cambridge, UK; 8 Signalling Research Center BIOSS, Freiburg, Germany; 9 Josep Carreras Leukemia Research Institute-Campus Clinic and Department of Biomedicine, School of Medicine, University of Barcelona, Barcelona, Spain; 10 Department of Hematology, Oncology and Stem Cell Transplantation, Faculty of Medical, University of Freiburg, Freiburg, Germany; 11 Institute for Research in Immunology and Cancer, University of Montreal, Montreal, Canada; 12 Catalan Institution for Research and Advanced Studies (ICREA), Barcelona, Spain; 13 Spanish Network for Cancer Research (CIBER-ONC)-ISCIII, Barcelona, Spain; 14 Department of Orthopedic Surgery, University Hospital of Cologne, Cologne, Germany; 15 Würzburg Institute of Systems Immunology, Max Planck Research Group at the Julius-Maximilians-Universität, Würzburg, Germany; 16 Helmholtz Institute for RNA-based Infection Research (HIRI), Helmholtz Centre for Infection Research (HZI), Würzburg, Germany

**Keywords:** Haematopoietic stem cells, Quiescence

## Abstract

Bone marrow haematopoietic stem cells (HSCs) are vital for lifelong maintenance of healthy haematopoiesis. In inbred mice housed in gnotobiotic facilities, the top of the haematopoietic hierarchy is occupied by dormant HSCs, which reversibly exit quiescence during stress. Whether HSC dormancy exists in humans remains debatable. Here, using single-cell RNA sequencing, we show a continuous landscape of highly purified human bone marrow HSCs displaying varying degrees of dormancy. We identify the orphan receptor GPRC5C, which enriches for dormant human HSCs. GPRC5C is also essential for HSC function, as demonstrated by genetic loss- and gain-of-function analyses. Through structural modelling and biochemical assays, we show that hyaluronic acid, a bone marrow extracellular matrix component, preserves dormancy through GPRC5C. We identify the hyaluronic acid–GPRC5C signalling axis controlling the state of dormancy in mouse and human HSCs.

## Abstract (web summary)

Zhang et al. use single-cell RNA sequencing and functional analyses to describe the hyaluronic acid–GPRC5C signalling axis as an essential component controlling the state of dormancy for human and mouse haematopoietic stem cells.

## Main

Haematopoietic stem cells (HSCs) sit at the top of a hierarchically organized system and can reconstitute the entire blood system^1,2^. Many seminal studies have demonstrated important mechanisms regulating human HSCs^3–9^. For instance, Dick and colleagues have recently shown that TFEB-mediated endolysosomal activity maintains long-term-HSC (LT-HSC) quiescence^10^. Cell cycle profiles have been used to further distinguish and characterize heterogeneity within human LT-HSCs^5,10–13^. This unique approach has allowed researchers to identify key mechanisms governing the HSC state, such as how a quiescent reservoir of human HSCs resists regenerative stress to preserve key stem cell features^12^. Still, most studies have largely focused on cord blood (CB) HSCs, which represent a more primitive population than their counterparts in the adult bone marrow (BM) and have a distinctive transcriptomic profile^10–12,14^. Delineating human HSCs in adult BM is critical to answering fundamental unresolved questions, including whether dormant HSCs (dHSCs) exist, how the stem cell state is maintained and how aberrant haematopoiesis such as leukaemia develops.

Using long-term label-retaining cell assays, two subsets of HSCs were previously identified in the mouse system: dHSCs and active HSCs (aHSCs)^15–17^. Both rest in a non-cycling quiescent state, but dHSCs are defined by a deeper state of quiescence and harbour the highest stem cell potential^17^. Maintaining dormancy minimizes stress from cellular respiration and DNA damage, contributing to HSC longevity and functionality^18^. Still, the concept of dormancy has only been established in artificial hygienic conditions such as gnotobiotic mouse facilities.

In this Article, we used single-cell RNA sequencing (scRNA-seq) to identify a population of stem cells resembling mouse dHSCs among human BM LT-HSCs. We found that GPRC5C enriches for dormancy and is required to maintain stem cell features. We identified the hyaluronic acid (HA)–GPRC5C signalling axis controlling HSC state.

## Results

### Dormancy in human BM stem cells

**Fig. 1:**
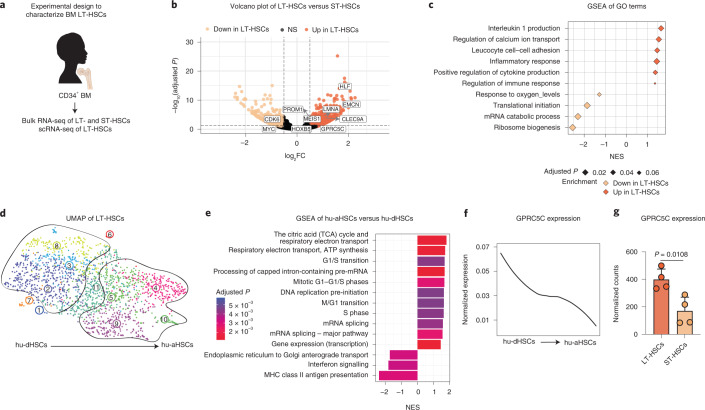
Identification of a highly dormant HSC population in human BM. **a**, Experimental design to characterize LT-HSCs from human BM donors. **b**, Volcano plot depicting DEGs between LT-HSCs and ST-HSCs from four BM donors. Benjamini–Hochberg (BH)-adjusted *P* values after Wald test. log_2_FC ≥0.5; adjusted *P* < 0.1. **c**, GSEA of GO terms in LT-HSCs compared with ST-HSCs. Normalized enrichment score (NES). **d**, UMAP of single-cell transcriptomes of human BM LT-HSCs from two donors highlighting VarID clusters. Numbers denote clusters. **e**, GSEA for genes that were differentially expressed between clusters 4, 5, 9 and 10 (hu-aHSCs) and 2, 3 and 8 (hu-dHSCs). BH-corrected *P* ≤ 0.05. **f**, Smoothed pseudo-temporal normalized expression profile of GPRC5C in LT-HSCs. Smoothed profiles were computed by local regression. **g**, Bulk RNA-seq data depicting normalized counts of GPRC5C in LT-HSCs and ST-HSCs. *n* = 4 biological replicates. Two-tailed *t*-test was performed. Data are presented as mean ± s.d. The *y* axis of the smoothed pseudo-temporal expression profiles indicates normalized expression. GSEA was performed with BH-adjusted *P* values after adaptive multi-level splitting Monte Carlo approach. LT-HSCs (CD34^+^CD38^−^CD45RA^−^CD90^+^CD49f^+^), ST-HSCs (CD34^+^CD38^−^CD45RA^−^CD90^−^CD49f^−^) and HSCs (CD34^+^CD38^−^). Source numerical data are available in source data. Source data

**Extended Data Fig. 1:**
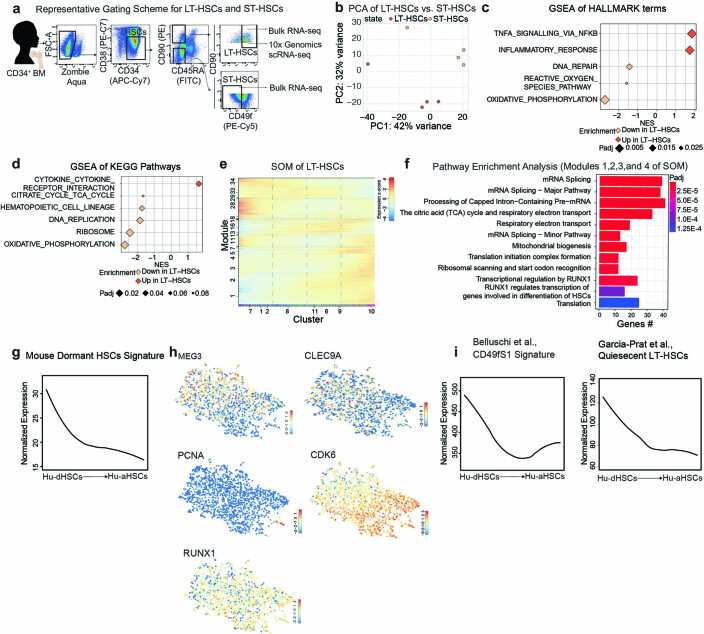
**a**. Representative FACS gating for HSCs (CD34^+^CD38^−^), ST-HSCs (CD34^+^CD38^−^CD90^−^CD45RA^−^CD49f^−^), and LT-HSCs (CD34^+^CD38^−^CD45RA^−^CD90^+^CD49f^+^) from CD34^+^ BM donors used for bulk RNA-seq and scRNA-seq. **b**. Principal component analysis (PCA) of RNA-seq data from LT-HSCs and ST-HSCs. Based on top 5,000 variable genes after variance-stabilization of DESeq2 Wald test. **c**. GSEA of Hallmark terms in LT-HSCs compared to ST-HSCs. **d**. GSEA of KEGG Pathways in LT-HSCs compared to ST-HSCs. **e**. SOM of LT-HSCs from scRNA-seq. **f**. Pathway enrichment analysis from modules 1, 2, 3, and 4 of SOM of LT-HSCs. Hypergeometric test. **g**. Smoothed pseudo-temporal normalized expression profile of *Mouse dormant HSCs* signature in LT-HSCs. Smoothed profiles were computed by local regression. **h**. LT-HSC expression UMAPs of the stem and activation associated genes. The colour bars indicate log_2_-normalized expression. **i**. Smoothed pseudo-temporal normalized expression profile of the *Cd49f1* signature from Bellushi et al., and *Quiescent LT-HSCs* from García-Prat et al., smoothed profiles were computed by local regression. Colour bars at the bottom of the SOMs show VarID clusters or RaceID clusters. The y-axis of the smoothed pseudo-temporal expression profiles indicates normalized expression. GSEA was performed with BH-adjusted p-values after adaptive multilevel splitting Monte Carlo approach.

To understand adult HSC regulation, we performed genome-wide transcriptional profiling of healthy BM LT-HSCs (CD34^+^CD38^−^CD45RA^−^CD90^+^CD49f^+^) and short-term HSCs (ST-HSCs; CD34^+^CD38^−^CD45RA^−^CD90^−^CD49f^−^) (Fig. 1a and Extended Data Fig. 1a, b). As expected, we detected upregulation of stem cell- and quiescence-associated genes (HLF and CLEC9A) and downregulation of cell cycle-related genes (CDK6 and MYC) in LT-HSCs compared with ST-HSCs (Fig. 1b and Supplementary Table 1). Gene set enrichment analysis (GSEA) showed enrichment for categories associated with immune function in LT-HSCs (Fig. 1c and Extended Data Fig. 1c,d) and metabolic activity in ST-HSCs (Fig. 1c and Extended Data Fig. 1c).

Next, we performed scRNA-seq of human BM LT-HSCs (Fig. 1a and Extended Data Fig. 1a) and found that cells formed a continuum (Fig. 1d)^19^. We pseudo-temporally ordered cells on the basis of their uniform manifold approximation and projection (UMAP) *x*-axis coordinate and then grouped pseudo-time profiles of all expressed genes into co-expression modules by applying a self-organizing map (SOM) (Extended Data Fig. 1e)^20^. Pathway enrichment analysis of the upregulated genes in modules 1, 2, 3 and 4, as defined by the SOM (corresponding to genes upregulated in clusters 9 and 10 of the UMAP), revealed an over-representation of terms associated with ‘translation’, suggesting these were more metabolically active HSCs (Extended Data Fig. 1f). GSEA of clusters 4, 5, 9 and 10 of the UMAP revealed an enrichment of cell cycle-related terms (Fig. 1e). Conversely, immune function-related terms were enriched in clusters 2, 3 and 8 of the UMAP. This pattern was consistent with processes previously observed when comparing human LT-HSCs versus ST-HSCs (Fig. 1c) and mouse dHSCs versus aHSCs^21^. We projected the ‘mouse dormant HSCs’ signature and found that clusters were organized along an ‘axis of dormancy’ (Extended Data Fig. 1g). Similar to mouse haematopoiesis, human HSC activation represented a continuum composed of intermediate cell states. The most dormant cells were located on the left-hand side of the UMAP (human dHSCs (hu-dHSCs)) expressing quiescence genes (MEG3 and CLEC9A), whereas the most active HSCs were on the right-hand side expressing activation-associated genes (human aHSCs (hu-aHSCs); CDK6 and PCNA; Fig. 1d and Extended Data Fig. 1g,h)^6^. Further, we found a positive enrichment of two recently published quiescent CB LT-HSC gene signatures when projected onto our axis of dormancy (Extended Data Fig. 1i)^10,11^.

### GPRC5C enriches for a quiescent stem cell population

**Fig. 2:**
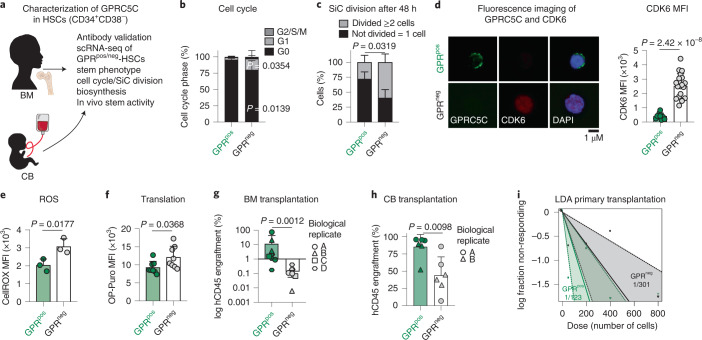
Characterization of GPRC5C as a marker of quiescent stem cells. **a**, Experimental design to characterize GPRC5C in human BM and CB. **b**, Fluorescence-activated cell sorting (FACS)-based analysis of cell cycle from BM GPRC5C^pos/neg^-HSCs. *n* = 5 biological replicates. **c**, Single-cell (SiC) division assay of GPRC5C^pos/neg^-HSCs from BM donors quantified after 48 h in vitro culture. *n* = 3 biological replicates. **d**, Left: fluorescence imaging of GPRC5C overlaid with intracellular CDK6 expression from BM HSCs. Right: quantification of the median fluorescence intensity (MFI) of CDK6 in GPRC5C^pos/neg^-HSCs from two BM donors. *n* = 10 GPRC5C^pos^ cells and *n* = 20 GPRC5C^neg^ cells. **e**, FACS measurement of CellROX Deep Red MFI in GPRC5C^pos/neg^-HSCs from BM donors. *n* = 3 biological replicates. **f**, FACS measurement of OP-Puro MFI in GPRC5C^pos/neg^-HSCs from BM donors. *n* = 8 biological replicates. **g**, GPRC5C^pos/neg^-HSCs from four BM donors were transplanted in NSBGW mice, which were killed at 36 weeks. Engraftment was assessed by percentage of human CD45-positive cells. *n* = 9 biological replicates. **h**, 800 GPRC5C^pos/neg^-HSCs from two CB donors were transplanted in NSBGW mice, which were killed at 24 weeks. Engraftment was assessed by percentage of human CD45-positive cells. *n* = 6 biological replicates. **i**, LDA to estimate the HSC frequency within GPRC5C^pos/neg^-HSCs from two CB donors at 24 weeks post-transplantation by extreme limiting dilution analysis (ELDA). All data presented as mean ± s.d. Statistical significance was determined by two-tailed *t*-test (**d**, **e**, **f** and **h**), two-way analysis of variance (ANOVA) (**b** and **c**), or Mann–Whitney *U* test (**g**). NS, not significant. *n* indicates number of replicates. For all experiments, at least two independent experiments were performed. Source numerical data are available in source data. Source data

**Fig. 3:**
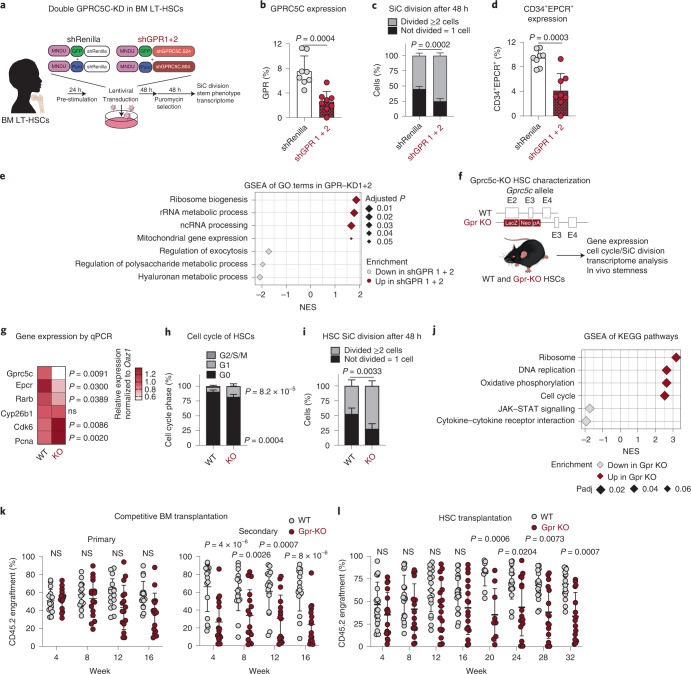
Loss of GPRC5C impairs stemness and quiescence. **a**, Experimental design to knock down and assess the impact of decreased GPRC5C levels in BM LT-HSCs. **b**, FACS validation of GPRC5C protein levels. *n* = 8 experiments. **c**, SiC division assay of GFP^+^ BM cells quantified after 48 h in vitro culture. *n* = 4 experiments for shGPR1 + 2 and *n* = 5 experiments for shRenilla. **d**, Surface expression of CD34^+^EPCR^+^ in GFP^+^ BM cells. *n* = 8 experiments. **e**, GSEA of GO terms in shGPR 1 + 2 compared with shRenilla control. **f**, Experimental design to assess functional impact of Gprc5c KO in mouse HSCs. Deletion of Gprc5c was achieved by the insertion of LacZ downstream of the translation initiation site in exon 2 of both alleles. **g**, Differential gene expression. Heat map representing median RNA expression from qPCR data (normalized to housekeeping gene Oaz1 and WT control). *n* = 8 biological replicates. **h**, FACS-based HSC cell cycle analysis with Ki-67 and DAPI. *n* = 5 biological replicates. **i**, SiC division assay of Gprc5c-KO and WT control HSCs quantified after 48 h in vitro culture. *n* = 4 biological replicates for Gpr KO and *n* = 5 biological replicates for WT. **j**, GSEA of KEGG pathways in Gprc5c-KO compared with WT control HSCs. **k**, PB analysis of competitive WBM chimaeras. CD45.2 cell percentage outcome in PB is presented. *n* = 16 biological replicates. **l**, PB analysis of HSC transplants. CD45.2 cell percentage outcome in PB is presented. *n* = 17 biological replicates for Gpr KO and *n* = 18 biological replicates for WT. All data presented as mean ± s.d. Statistical significance was determined by two-tailed *t*-test (**b**, **d** and **g**) or two-way ANOVA (**c**, **h**, **I**, **k** and **l**). *n* indicates number of replicates. GSEA was performed with BH-adjusted *P* values after adaptive multi-level splitting Monte Carlo approach. For all experiments, at least two independent experiments were performed. At least two human BM donors were used (**b**–**d**). Source numerical data are available in source data. Source data

**Extended Data Fig. 2:**
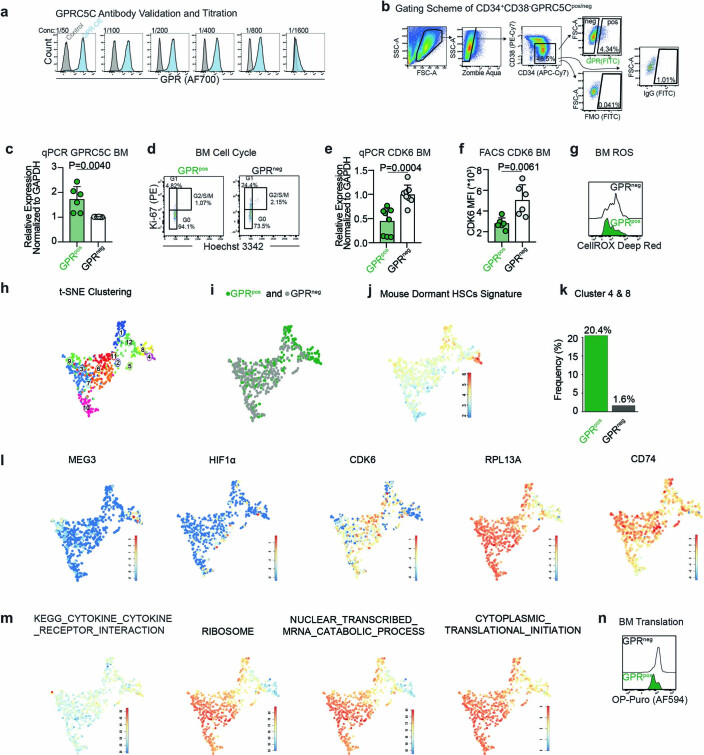
**a**. Titration of monoclonal human GPRC5C antibody. HEK293T transfected with overexpressing GPRC5C or control plasmid and stained with decreasing concentration (left to right) of the GPRC5C antibody. **b**. Representative FACS scheme for gating of GPRC5C^pos/neg^-HSCs. GPRC5C^pos^ cells are identified using a fluorescence minus one (FMO) or IgG control. **c**. qPCR validation of *GPRC5C* RNA expression from BM GPRC5C^pos/neg^-HSCs. Normalized to housekeeping gene *GAPDH* and GPRC5C^neg^. *n* = 6 biological replicates. **d**. Representative FACS plots of cell cycle profiles from BM GPRC5C^pos/neg^-HSCs. **e**. qPCR of *CDK6* expression from GPRC5C^pos/neg^-HSCs. Normalized to *GAPDH* gene and then GPRC5C^neg^-HSCs. *n* = 8 biological replicates. **f**. FACS measurement of CDK6 MFI in BM GPRC5C^pos/neg^-HSCs. *n* = 6 biological replicates. **g**. Representative FACS histogram of CellROX Deep Red MFI in BM GPRC5C^pos/neg^-HSCs. **h**. t-SNE representation of the clusters from BM GPRC5C^pos/neg^-HSCs. **i**. t-SNE depicting BM GPRC5C^pos/neg^-HSCs. **j**. Projection of the t-SNE of the *Mouse Dormant HSCs* signature. The colour bars indicate log_2_-normalized expression. **k**. The enrichment of GPRC5C^pos^-HSCs in clusters 4 and 8 is 20.4% compared to 1.6% for GPRC5C^neg^-HSCs. **l**. t-SNE of stem- and activation-associated genes. The colour bars indicate log_2_-normalized expression. **m**. t-SNE of KEGG and GO pathways. The colour bars indicate log_2_-normalized expression. **n**. Representative FACS histogram of OP-Puro MFI in BM GPRC5C^pos/neg^-HSCs. All data presented as mean ± SD. Statistical significance was determined using two-tailed t-test (c, e, f) and at least two independent experiments were performed. ns, not significant. *n* indicates number of replicates. Source numerical data are available in source data. Source data

To characterize dormancy in human stem cells, we screened for potential cell surface receptors. We decided to focus on GPRC5C because (1) its expression was enriched within hu-dHSCs (Fig. 1f), (2) it showed higher expression in LT-HSCs compared with ST-HSCs (Fig. 1g), and (3) GPRC5C had not been studied in humans^21^. Using a monoclonal human GPRC5C antibody, we investigated the role of GPRC5C in quiescence (Extended Data Fig. 2a–c). We analysed the cell cycle profile and found that GPRC5C^pos^-HSCs (CD34^+^CD38^−^) resided almost exclusively in the G_0_ phase, while GPRC5C^neg^-HSCs were detected in both the G_0_ and G_1_ phases (Fig. 2a,b and Extended Data Fig. 2d). In vitro single-cell division analysis showed that GPRC5C^neg^-HSCs were twice as likely to undergo division compared with GPRC5C^pos^-HSCs (Fig. 2c). Consistent with CDK6 levels being linked to a quiescent but cell-cycle-primed state^6^, GPRC5C^pos^-HSCs expressed lower levels of CDK6 (Fig. 2d and Extended Data Fig. 2e,f). Using CellROX, we found lower reactive oxygen species (ROS) levels within the GPRC5C^pos^-HSCs (Fig. 2e and Extended Data Fig. 2g). We next performed index sorting of BM GPRC5C^pos/neg^-HSCs for mCEL-Seq2 scRNA-seq^20,22^ (Extended Data Fig. 2h). Notably, only weak GPRC5C RNA expression was detected, and downstream analyses relied on the index sorting to identify GPRC5C^pos/neg^-HSCs (Extended Data Fig. 2i). The ‘mouse dormant HSCs’ signature was enriched in clusters 4 and 8, which were mainly represented by GPRC5C^pos^-HSCs (Extended Data Fig. 2j,k). Similarly, we found enrichment of stem-cell-related genes (MEG3 and HIF1a) complemented by lower expression of activation-related genes (CDK6 and CD74 (ref. ^23^); Extended Data Fig. 2l). At the process level, immune-related terms were enriched, while translation-related processes were poorly represented as also shown by lower OP-Puro incorporation (a measurement of de novo protein synthesis)^24^ in GPRC5C^pos^-HSCs (Fig. 2f and Extended Data Fig. 2m,n).

**Extended Data Fig. 3:**
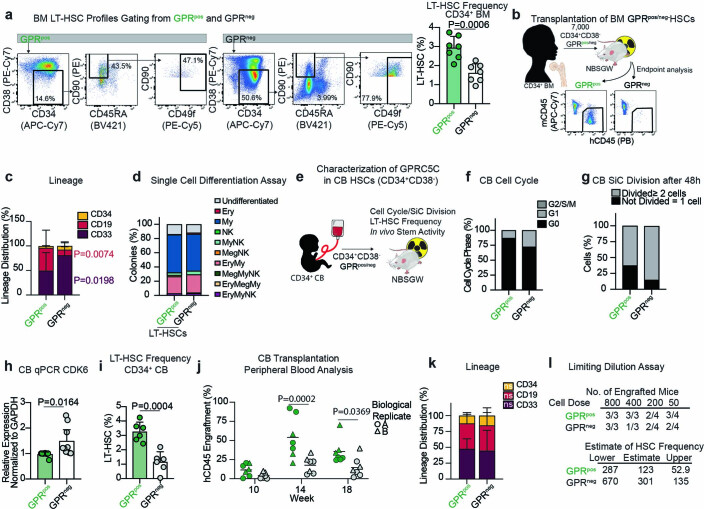
**a**. Representative FACS gating scheme of LT-HSCs within GPRC5C^pos/neg^ population from CD34^+^ BM donors. Frequency of LT-HSCs within GPRC5C^pos/neg^ cells from CD34^+^ BM donors. *n* = 7 biological replicates. **b**. Experimental design to assess the *in vivo* reconstitution ability of BM GPRC5C^pos/neg^-HSCs. **c**. Lineage distribution of GPRC5C^pos/neg^-HSCs. Data presented refer to the distribution of myeloid (CD33), B-lymphoid (CD19) and immature surface phenotype (CD34). *n* = 9 biological replicates. **d**. Percentage of determined colony types derived from single BM GPRC5C^pos/neg^ -LT-HSCs following culture under differentiating conditions for three weeks. *n* = 2 biological replicates. **e**. Experimental design to characterize GPRC5C in human CB. **f**. FACS analysis of cell cycle from CB GPRC5C^pos/neg^-HSCs. *n* = 2 biological replicates. **g**. SiC division assay of GPRC5C^pos/neg^-HSCs from CB donors quantified after 48 hours *in vitro* culture. *n* = 2 biological replicates. **h**. qPCR of *CDK6* expression from CB GPRC5C^pos/neg^-HSCs. Normalized to housekeeping gene *GAPDH* and GPRC5C^pos^-HSCs. *n* = 9 biological replicates. **i**. Frequency of LT-HSCs within GPRC5C^pos/neg^ cells from CD34^+^ CB donors. *n* = 6 biological replicates. **j**. Assessment of reconstitution dynamics. Engraftment was assessed by percentage of human CD45 cells in the PB. *n* = 6 biological replicates. **k**. Lineage contribution of CB GPRC5C^pos/neg^-HSCs. Data presented refer to the distribution of myeloid (CD33), B-lymphoid (CD19), and immature surface phenotype (CD34). *n* = 6 biological replicates. **l**. Table showing the number of cells injected per dose, number of mice per dose, and the number of engrafted mice per dose. These data were used to calculate the HSC frequency seen in Fig. 2i. All data presented as mean ± SD. Statistical significance was determined using two-tailed t-test (a, h, i, j) or two-way ANOVA (c and k) and at least two independent experiments were performed. ns, not significant. *n* indicates number of replicates. Source numerical data are available in source data. Source data

Phenotypically, GPRC5C^pos^ cells in CD34^+^-enriched BM possessed twice the LT-HSCs compared with GPRC5C^neg^ (Extended Data Fig. 3a). Functionally, in vivo reconstitution levels of BM GPRC5C^pos^-HSCs were higher compared with GPRC5C^neg^-HSCs (Fig. 2g and Extended Data Fig. 3b). We observed an increased frequency of CD19^+^-lymphoid cells in GPRC5C^pos^-HSC-engrafted animals, likely reflecting the larger grafts (Fig. 2g and Extended Data Fig. 3c). Single-cell differentiation assay of GPRC5C^pos/neg^-LT-HSCs showed no differences in the types of colonies produced (Extended Data Fig. 3d).

GPRC5C^pos^-HSCs from CB mimicked the properties of BM-derived GPRC5C^pos^-HSCs, including a higher frequency of G_0_ cells, delayed entry into the cell cycle, lower CDK6 expression and enrichment of phenotypic LT-HSCs compared with CB GPRC5C^neg^-HSCs (Extended Data Fig. 3e–i). Functionally, peripheral blood (PB) and BM analysis from in vivo transplantation revealed that CB GPRC5C^pos^-HSCs had higher reconstitution capacity, with no lineage bias (Fig. 2h and Extended Data Fig. 3j,k). Limiting dilution analysis (LDA) revealed CB GPRC5C^pos^-HSCs contained twice as many HSCs compared with CB GPRC5C^neg^-HSCs (Fig. 2i and Extended Data Fig. 3l)

In summary, GPRC5C^pos^-HSCs are highly quiescent, possess low biosynthetic activity and have high long-term reconstitution capabilities.

### Loss of GPRC5C disturbs quiescence and stemness

**Extended Data Fig. 4:**
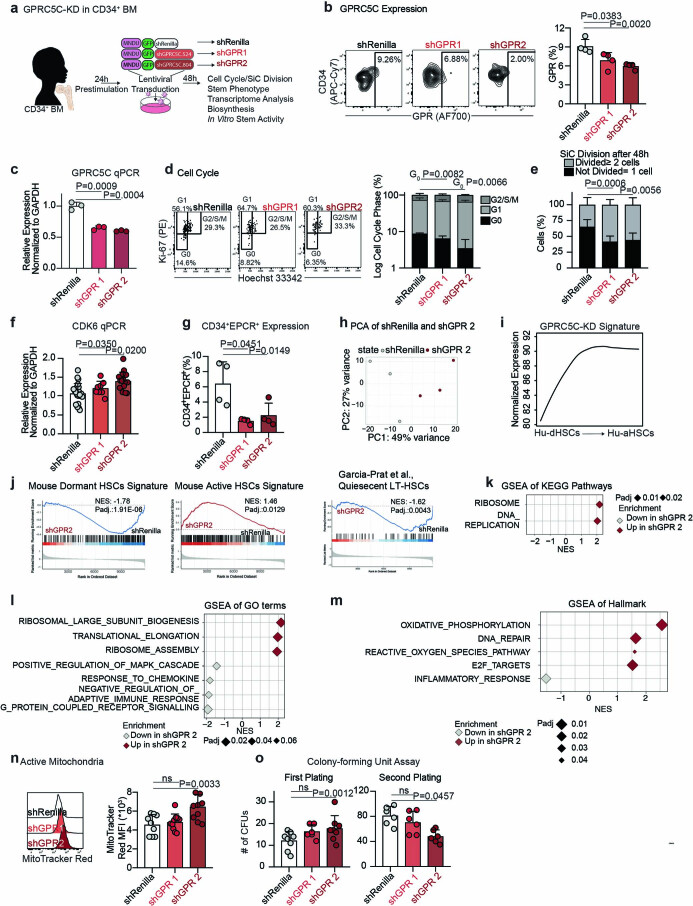
**a**. Experimental design to knockdown and assess the impact of decreased GPRC5C levels in CD34^+^ BM. **b**. FACS validation of GPRC5C protein levels quantified in GFP^+^ BM cells. *n* = 4 experiments. **c**. qPCR of *GPRC5C* expression in GFP^+^ BM cells. Normalized to housekeeping gene *GAPDH* and shRenilla. *n* = 3 experiments. **d**. Cell cycle FACS analysis on GFP^+^ BM cells. Cell cycle phase is represented on log10 scale. *n* = 4 experiments. **e**. SiC division assay of GFP^+^ BM cells quantified after 48 hours *in vitro* culture. *n* = 4 experiments. **f**. qPCR of *CDK6* expression in GFP^+^ BM cells. Normalized to housekeeping gene *GAPDH* and shRenilla. *n* = 8 experiments. **g**. Surface expression of CD34^+^EPCR^+^ in GFP^+^ BM cells. *n* = 4 experiments. **h**. PCA of RNA-seq data from shGPR 2 and shRenilla control. Based on top 5,000 variable genes. log_2_FC threshold ≥ 0.5, padj. < 0.1. **i**. Smoothed pseudo-temporal expression profile of the *GPR-KD* signature in LT-HSCs. Smoothed profiles were computed by local regression. The y-axis of the smoothed pseudo-temporal expression profiles indicates normalized expression. **j**. GSEA of *Mouse Dormant HSCs, Mouse Active HSCs*, and *Quiescent LT-HSCs* from García-Prat et al., signature in shGPR 2 and shRenilla control. **k**. GSEA of KEGG pathways in shGPR 2 compared to shRenilla control. **l**. GSEA of GO terms in shGPR 2 compared to shRenilla control. **m**. GSEA of Hallmarks terms in shGPR 2 compared to shRenilla control. **n**. FACS measurement of MitoTracker Red MFI in GFP^+^ BM. *n* = 10 experiments. **o**. CFU assay of GFP^+^ cells from GPRC5C-KD and shRenilla control. Primary plating *n* = 8 experiments, except *n* = 6 experiments for shGPR1. Secondary plating *n* = 6 experiments. All data presented as mean ± SD. Statistical significance was determined using two-tailed t-test, except d and e (two-way ANOVA). GSEA performed with BH-adjusted p-values after adaptive multilevel splitting Monte Carlo approach. ns, not significant. *n* indicates the number of biological replicates. For all experiments, at least two independent experiments were performed. At least two human BM donors were used. Source numerical data are available in source data. Source data

To explore the functionality of GPRC5C, we transduced human CD34^+^ BM cells with a GPRC5C-targeting short hairpin RNA vector (Extended Data Fig. 4a–c). GPRC5C knockdown (KD) resulted in decreased cells in G_0_ phase, a faster division rate and increased CDK6 expression (Extended Data Fig. 4d–f). CD34^+^EPCR^+^, a reliable marker of ex vivo expanded HSCs^3^, was reduced upon GPRC5C KD (Extended Data Fig. 4g). We performed RNA-seq analysis, projected the gene signature of GPRC5C-KD cells onto our axis of dormancy (Fig. 1d, scRNA-seq of LT-HSCs), and found it was enriched for hu-aHSCs genes (Extended Data Fig. 4h,i). Similarly, GPRC5C KD upregulated genes associated with ‘mouse active HSCs’, and downregulated genes associated with ‘mouse dormant HSCs’ and ‘quiescent LT-HSCs’ from García-Prat et al.^10^ (Extended Data Fig. 4j). GSEA indicated that ‘oxidative phosphorylation’ and ‘reactive oxygen species pathway’ were enriched in GPRC5C-KD cells, which was corroborated by MitoTracker Red, an indicator of mitochondrial activity (Extended Data Fig. 4k–n). We observed upregulation of translation-related terms and downregulation of immune-related processes upon GPRC5C KD (Extended Data Fig. 4k–m). Functionally, we performed in vitro serial colony-forming unit (CFU) assays. Primary plating of GPRC5C-KD cells exhibited increased colony-forming abilities, whereas secondary plating produced fewer colonies compared with control (Extended Data Fig. 4o). This is consistent with the notion that GPRC5C-KD cells have enhanced progenitor activity but reduced stem-like properties.

To validate our analysis on a purer HSC population and improve KD efficiency, we used BM LT-HSCs and targeted *GPRC5C* using two short hairpin RNAs (Fig. 3a). Consistent with our previous findings, we found accelerated in vitro single-cell division and a decrease in immature CD34^+^EPCR^+^ cells upon GPRC5C KD (Fig. 3b–d). GSEA of GPRC5C-KD cells showed enrichment of terms related to biogenesis and processing pathways, indicating an enhanced metabolically active state in GPRC5C-KD HSCs (Fig. 3e).

**Extended Data Fig. 5:**
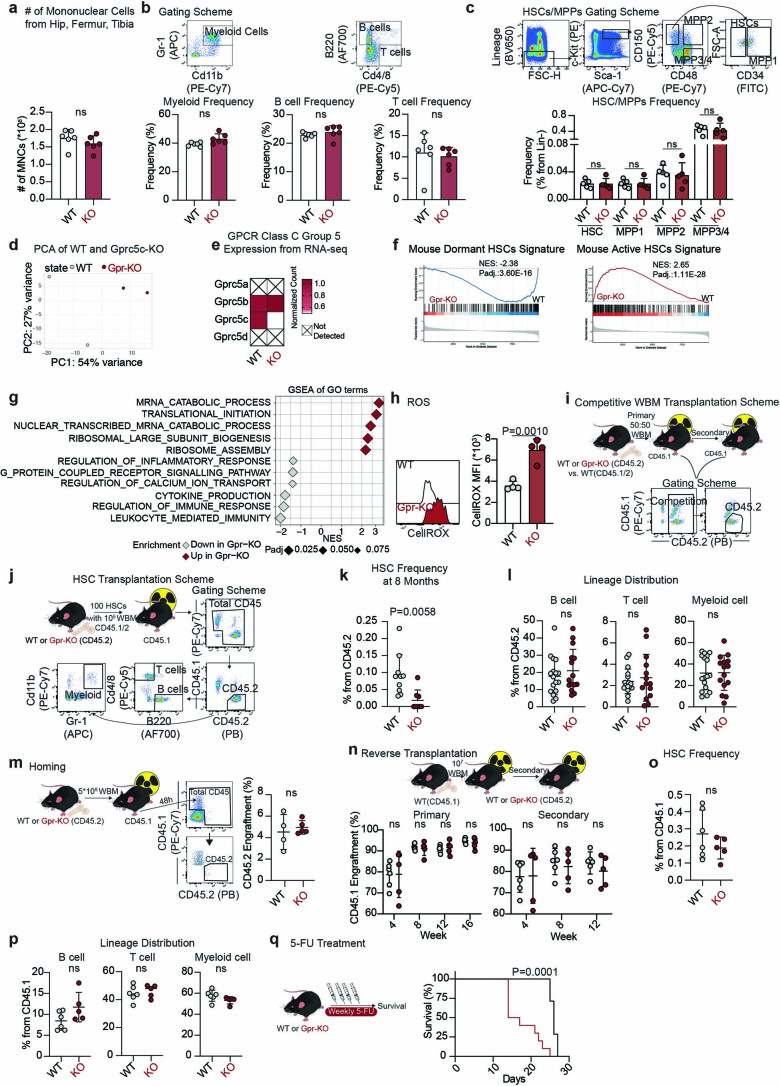
**a**. Absolute number of mononuclear cells (MNC) isolated from hip, femur, and tibia. *n* = 6 biological replicates. **b**. FACS gating scheme for myeloid, B cells and T cells. Frequency of described populations from Gprc5c-KO and WT control BM. *n* = 6 biological replicates. **c**. Upper panel: FACS gating scheme for HSCs (LSK-CD150^+^CD48^+^CD34^−^), MPP1 (LSK-CD150^+^CD48^+^CD34^+^), MPP2 (LSK-CD150^+^CD48^+^), and MPP3/4 (LSK-CD150^−^CD48^+^). Lower panel: frequency of described populations from Gprc5c-KO and WT control BM. *n* = 5 biological replicates. **d**. PCA of RNA-seq data from Gprc5c-KO and WT control HSCs. Based on top 5,000 variable genes. log_2_FC threshold ≥ 0.5, padj.<0.1. **e**. Differential gene expression. Heatmap representing mean of normalized counts (normalized to WT). *n* = 2 biological replicates. **f**. GSEA of gene signatures in Gprc5c-KO and WT control HSCs. **g**. GSEA of GO terms in Gprc5c-KO compared to WT control HSCs. **h**. FACS measurement of CellROX Deep Red MFI in Gprc5c-KO compared to WT control HSCs. *n* = 4 biological replicates. **i**. Experimental design for competitive WBM transplantation. 50:50 ratio transplant of CD45.1/2 WT: CD45.2 WT or Gprc5c-KO WBM into CD45.1 recipient mice. Representative FACS gating scheme to quantify engraftment. **j**. Experimental design for HSC transplantation and FACS gating scheme to quantify engraftment and lineage distribution. **k**. Endpoint frequency of BM HSCs in CD45.2 cells of Gprc5c-KO (*n* = 8 biological replicates) and WT control (*n* = 10 biological replicates). **l**. Lineage distribution of BM Gprc5c-KO (*n* = 14 biological replicates) and WT control (*n* = 16 biological replicates) from HSC transplantations. **m**. Left panel: experimental design and FACS gating scheme of homing assay. Right panel: frequency of CD45.2 cells engraftment 48 hours post transplantation. *n* = 4 biological replicates. **n**. Upper panel: experimental design for reverse chimeras. Lower panel: PB analysis of reverse chimeras. Engraftment is represented by CD45.1 cell percentage engraftment. *n* = 5 biological replicates for Gpr-KO and *n* = 6 biological replicates for WT mice. **o**. Endpoint frequency of BM HSCs from CD45.1 cells transplanted in Gprc5c-KO (*n* = 5 biological replicates) and WT control (*n* = 6 biological replicates) mice. **p**. Lineage distribution of BM CD45.1 cells transplanted in Gprc5c- KO (*n* = 5 biological replicates) and WT control (*n* = 6 biological replicates) mice. **q**. Survival curve of Gprc5c-KO (*n* = 10 biological replicates) and WT control (*n* = 7 biological replicates) mice treated with 5-FU. All data presented as mean ± SD. Statistical significance was determined using two-tailed t-test, except panel q: Mantel-Cox test. GSEA performed with BH-adjusted p-values after adaptive multilevel splitting Monte Carlo approach. ns, not significant. *n* indicates the number of biological replicates. Source numerical data are available in source data. Source data

Since GPRC5C expression is conserved between mice and humans, we used Gprc5c-knockout (KO) mice to perform experiments that are challenging or not feasible using primary human samples (Fig. 3f)^25^. Gprc5c-KO mice showed no differences in the frequency of phenotypic HSCs (Lineage^−^Sca1^+^cKit^+^[LSK]CD150^+^ CD48^−^CD34^−^), multipotent progenitors (MPP) or differentiated blood cells compared with controls (Extended Data Fig. 5a–c). However, Gprc5c-KO HSCs showed downregulation of dormancy-associated genes (Rarb and Epcr) and upregulation of cell cycle-associated genes (Pcna and Cdk6) (Fig. 3g). Examination of the cell cycle profiles of Gprc5c-KO HSCs revealed a slight decrease of cells in the G_0_ phase (Fig. 3h). In vitro single-cell cultivation of HSCs confirmed that Gprc5c KO had an accelerated division rate compared with wild-type (WT) control (Fig. 3i). Transcriptome analysis of Gprc5c-KO and WT HSCs indicated no compensatory upregulation of other members of GPCR class C group 5 (Extended Data Fig. 5d,e). GSEA analysis revealed downregulation of the ‘mouse dormant HSCs’ signature and upregulation of the ‘mouse active HSCs’ signature in Gprc5c-KO HSCs (Extended Data Fig. 5f). Subsequently, processes associated with ‘translation’ and ‘cell cycle’ were significantly enriched in Gprc5c-KO HSCs (Fig. 3j and Extended Data Fig. 5g). CellROX staining also revealed increased ROS levels in Gprc5c-KO HSCs (Extended Data Fig. 5h). These data indicate that loss of Gprc5c primes HSCs towards activation.

Functionally, we generated whole BM (WBM) competitive and HSC chimaeras (Extended Data Fig. 5i,j). From secondary WBM transplantations, we observed a significant reduction in the competitive engraftment potential of Gprc5c-KO cells compared with WT control (Fig. 3k). Similarly, we detected reduced reconstitution capabilities in Gprc5c-KO HSCs, but no changes in lineage distribution (Fig. 3l and Extended Data Fig. 5k,l). Possible bias from the initial engraftment and niche-specific effects were excluded by performing mobilization and reverse chimaera experiments (Extended Data Fig. 5m–p). Lastly, it has been shown that dHSCs are essential for proliferation-related stress^18,26^. Consistent with this, we found that Gprc5c-KO mice were more susceptible to serial injections of the chemotherapeutic agent fluorouracil (5-FU) (Extended Data Fig. 5q). Together, our results suggest that loss of Gprc5c leads to a loss of quiescence, self-renewal, and ultimately, an impaired ability to cope with proliferative stress conditions.

### Enhanced quiescence and stemness by overexpressing GPRC5C

**Fig. 4:**
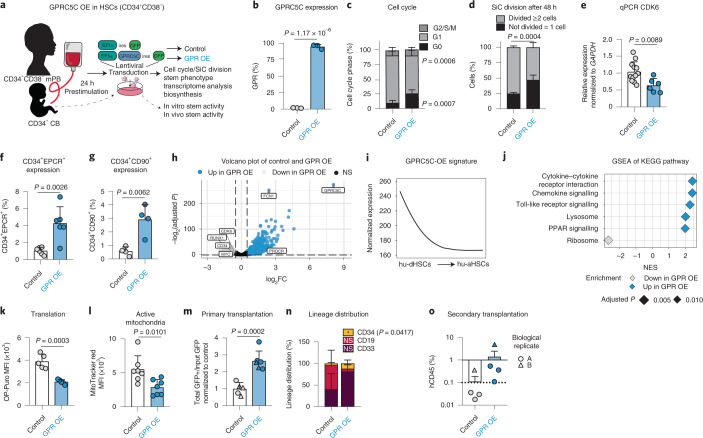
GPRC5C OE enhances stemness. **a**, Experimental design of GPRC5C OE in mPB and CB. Internal ribosomal entry site (ires). **b**, FACS validation of GPRC5C OE at protein level. *n* = 3 experiments. **c**, FACS-based cell cycle analysis of GPRC5C OE and control. *n* = 6 experiments. **d**, SiC division assay of GPRC5C OE and control quantified after 48 h in vitro culture. *n* = 4 experiments. **e**, qPCR of CDK6 expression. Normalized to housekeeping gene GAPDH and control. *n* = 6 experiments for GPRC5C OE and *n* = 12 experiments for control. **f**, Surface expression of CD34^+^EPCR^+^ in GPRC5C OE and control. *n* = 6 experiments. **g**, Surface expression of CD34^+^CD90^+^ in GPRC5C OE and control. *n* = 4 experiments. **h**, Volcano plot depicting DEGs between GPRC5C OE and control. Key genes associated with dormancy and stemness are highlighted. BH-adjusted *P* values after Wald test. log_2_FC ≥0.5; adjusted *P* < 0.1. **i**, Smoothed pseudo-temporal expression profile of the GPRC5C-OE signature in LT-HSCs. Smoothed profiles were computed by local regression. The *y* axis indicates normalized expression. **j**, GSEA of KEGG pathways in GPRC5C OE compared with control. Performed with BH-adjusted *P* values after adaptive multi-level splitting Monte Carlo approach. **k**, FACS measurement of OP-Puro MFI in GPRC5C OE and control. *n* = 5 experiments. **l**, FACS measurement of MitoTracker Red MFI in GPRC5C OE and control. *n* = 7 experiments. **m**, Transplantation of GPRC5C OE and control in CB cells from two biological replicates. Engraftment was measured from BM and calculated by the ratio of the total percentage GFP^+^hCD45^+^ to the percentage input GFP post-transplantation and normalized to control. *n* = 5 control and *n* = 6 GPR-OE biological replicates. **n**, Lineage distribution of GPRC5C OE and control in CB cells. Distribution of myeloid (CD33), B-lymphoid (CD19) and immature surface phenotype (CD34) are presented. *n* = 5 control and *n* = 6 GPR-OE biological replicates. **o**, Secondary transplantation of 150,000 GFP^+^hCD45^+^ cells. *n* = 4 biological replicates. All data presented as mean ± s.d. Statistical significance was determined using two-tailed *t*-test (**b**, **e**, **f**, **g**, **l**, **m**, **n** and **k**) or two-way ANOVA (**c** and **d**). *n* indicates number of replicates. For all experiments, at least two independent experiments were performed. At least two human mPB or CB donors were used. Source numerical data are available in source data. Source data

**Extended Data Fig. 6:**
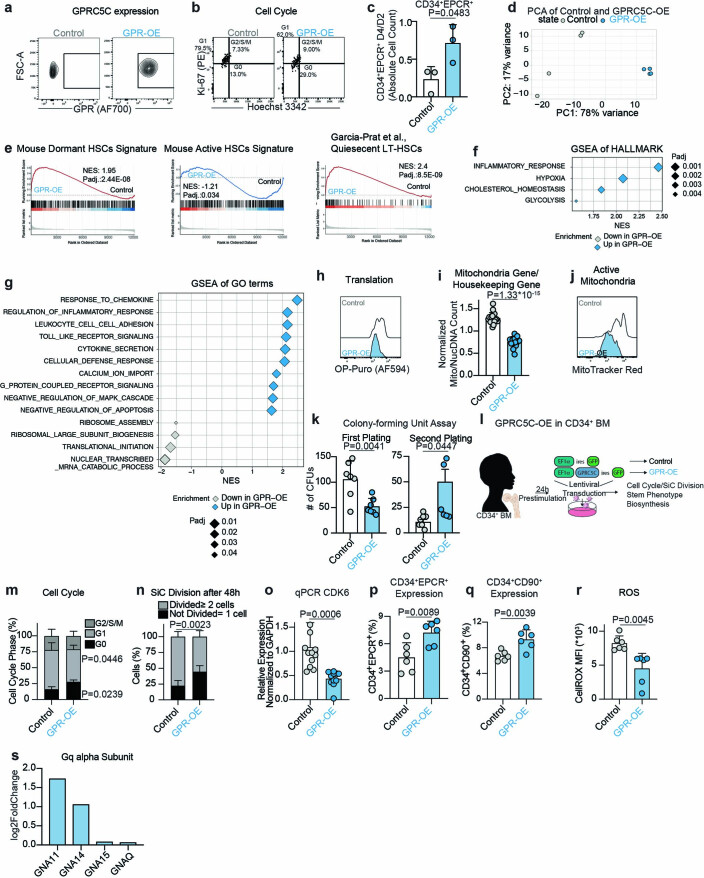
**a**. Representative FACS plot for validation of GPRC5C-OE in GFP^+^ mPB cells. **b**. Representative FACS plot of cell cycle profiles from GFP^+^ mPB cells. **c**. Ratio of the absolute number of CD34^+^EPCR^+^ cells from day 4 compared to day 2 *in vitro*. *n* = 3 experiments. **d**. PCA of RNA-seq data from GPRC5C-OE and control. Based on top 5,000 variable genes. log_2_FC threshold ≥ 0.5, padj. < 0.1. **e**. GSEA of quiescent signatures in GPRC5C-OE compared to control. **f**. GSEA of Hallmarks terms in GPRC5C-OE compared to control. **g**. GSEA of GO terms in GPRC5C-OE compared to control. **h**. FACS histogram of OP-Puro MFI in GPRC5C-OE and control. **i**. RNA-seq data showing ratio read count of mitochondria genes normalized to housekeeping genes (*GAPDH*, *ACTB*, *PPIA*). *n* = 19 genes averaged from 4 technical replicates. **j**. FACS histogram of MitoTracker Red MFI in GPRC5C-OE and control. **k**. CFU assay of GFP^+^ cells from GPRC5C-OE and control. *n* = 7 experiments, except for secondary plating GPR-OE, where *n* = 6 experiments. **l**. Experimental design to overexpress and assess the impact of the gain of GPRC5C expression in BM CD34 + . **m**. FACS-based cell cycle analysis of GPRC5C-OE and control. *n* = 6 experiments. **n**. SiC division assay of GPRC5C-OE and control quantified after 48 hours *in vitro* culture. *n* = 6 experiments. **o**. qPCR of *CDK6* expression. Normalized to housekeeping gene *GAPDH* and control. *n* = 12 experiments for WT and *n* = 11 experiments for GPR-OE. **p**. Surface expression of CD34^+^EPCR^+^ in GPRC5C-OE and control. *n* = 6 experiments. **q**. Surface expression of CD34^+^CD90^+^ in GPRC5C-OE and control. *n* = 4 experiments. **r**. FACS measurement of CellROX Deep Red MFI in GPRC5C-OE and control. *n* = 6 experiments. **s**. Log_2_FC of proteins of Gq alpha subunit. All data presented as mean ± SD. Statistical significance was determined using two-tailed t-test, except m and n (two-way ANOVA). GSEA performed with BH-adjusted p-values after adaptive multilevel splitting Monte Carlo approach. ns, not significant. *n* indicates the number of biological replicates. For all experiments, at least two independent experiments were performed. At least two human BM donors were used. Source numerical data are available in source data. Source data

Next, we engineered GPRC5C overexpression (OE) in human CD34^+^CD38^−^ mobilized peripheral blood (mPB) cells (Fig. 4a,b and Extended Data Fig. 6a). GPRC5C OE led to enhanced quiescence as indicated by an increase of G_0_ cells, a slower first division rate and decreased CDK6 expression (Fig. 4c–e and Extended Data Fig. 6b). As expected, we observed an activation of HSCs upon culture, in contrast to freshly isolated HSCs, which are highly quiesecent^10^ (Fig. 2c). We detected maintenance of CD34^+^CD90^+^ and CD34^+^EPCR^+^ cells in GPRC5C-OE cells, consistent with enhanced HSC functionality (Fig. 4f,g and Extended Data Fig. 6c).

Transcriptome analysis revealed downregulation of genes linked to activation or cell cycle (CDK6 and MYC) and upregulation of stemness-associated genes (PROCR) in GPRC5C-OE cells (Fig. 4h and Extended Data Fig. 6d). Interrogation of expression of the upregulated genes from GPRC5C-OE cells in the scRNA-seq data of LT-HSCs revealed that their expression was enriched in the hu-dHSC population (Fig. 1d and Fig. 4i). Similarly, GSEA revealed enrichment of genes related to ‘mouse dormant HSCs’ and ‘quiescent LT-HSCs’ from García-Prat et al.^10^ (Extended Data Fig. 6e). Kyoto Encyclopedia of Genes and Genomes (KEGG) and Gene Ontology (GO) analysis showed that terms associated with inflammatory signalling were enriched while ‘ribosome’ and ‘translational initiation’ were downregulated in GPRC5C-OE cells. The decrease in de novo translation was confirmed using OP-Puro (Fig. 4j,k and Extended Data Fig. 6f–h). We probed the expression of mitochondrial genes in the transcriptome and observed reduced mitochondrial DNA copy number (Extended Data Fig. 6i). In addition, we found decreased mitochondria potential in the GPRC5C-OE cells, as indicated by MitoTracker Red analysis (Fig. 4l and Extended Data Fig. 6j).

Serial CFU assay revealed that GPRC5C-OE cells had enhanced serial in vitro self-renewal capacity, which was confirmed by in vivo transplantation (Fig. 4m and Extended Data Fig. 6k). Lineage distribution revealed no difference, but GPRC5C OE showed a slight increase in the frequency of CD34^+^ cells, consistent with enhanced HSC self-renewal capabilities (Fig. 4n). In secondary transplantations, all four GPRC5C-OE mice (compared with only one of four control mice) met the requirement of positive human engraftment (≥0.1% hCD45 cells) (Fig. 4o). In agreement, GPRC5C OE in BM cells also resulted in increased quiescence, lower ROS and enrichment of phenotypic HSC surface marker expression (Extended Data Fig. 6l–r).

We thus observed that ectopic GPRC5C reduced biosynthesis, but increased quiescence and enhanced the reconstitution ability of HSCs.

### HA activates GPRC5C signalling

**Fig. 5:**
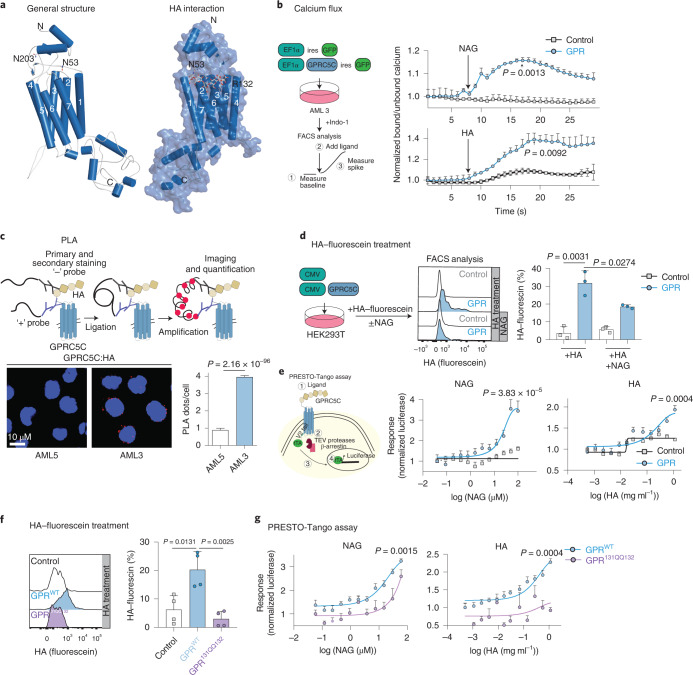
HA activates GPRC5C signalling. **a**, Left: structural modelling of GPRC5C. Right: docked pose of the HA at the 7TM region of GPRC5C. **b**, Experimental design to determine intracellular calcium concentrations upon interaction with putative ligands. Normalized baseline calcium followed by flux in response to HA or NAG in control and GPRC5C-OE conditions in AML3. *n* = 3 experiments. **c**, PLA between GPRC5C and HA, showing a distance smaller than 80 nm in AML3 cells but not AML5 cells. Plot representative of one of three independent experiments. Each independent experiment was performed in two technical replicates. *n* = 2250 cells for AML5 and *n* = 2091 cells for AML3. Presented as mean ± s.e.m. **d**, HA–fluorescein treatment of HEK293T cells expressing GPRC5C and control. FACS plot representing HA retention in each condition. *n* = 3 experiments. **e**, Left: schematic representation of PRESTO-Tango assay. Right: concentration–response curve of PRESTO-Tango construct for GPRC5C and control vector treated with NAG and HA in HTLA cells. Normalized to luciferase signal at the lowest concentration. *n* = 6 experiments for NAG and *n* = 5 experiments for HA. **f**. HA–fluorescein treatment of HEK293T cells expressing GPRC5C^WT^, GPRC5C^131QQ132^ and control. FACS plot representing HA retention in each condition. *n* = 4 experiments. **g**, Concentration–response curve of PRESTO-Tango construct for GPRC5C WT and site-specific mutant (GPRC5C^131QQ132^) at predicted HA interaction site in HTLA cells. *n* = 6 experiments. All data presented as mean ± s.d. unless otherwise stated. Statistical significance was determined using unpaired two-tailed *t*-test (**b**, **c**, **d**, **e**, **f** and **g**). *n* indicates number of replicates. For all experiments, at least two independent experiments were performed. Source numerical data are available in source data. Source data

**Extended Data Fig. 7:**
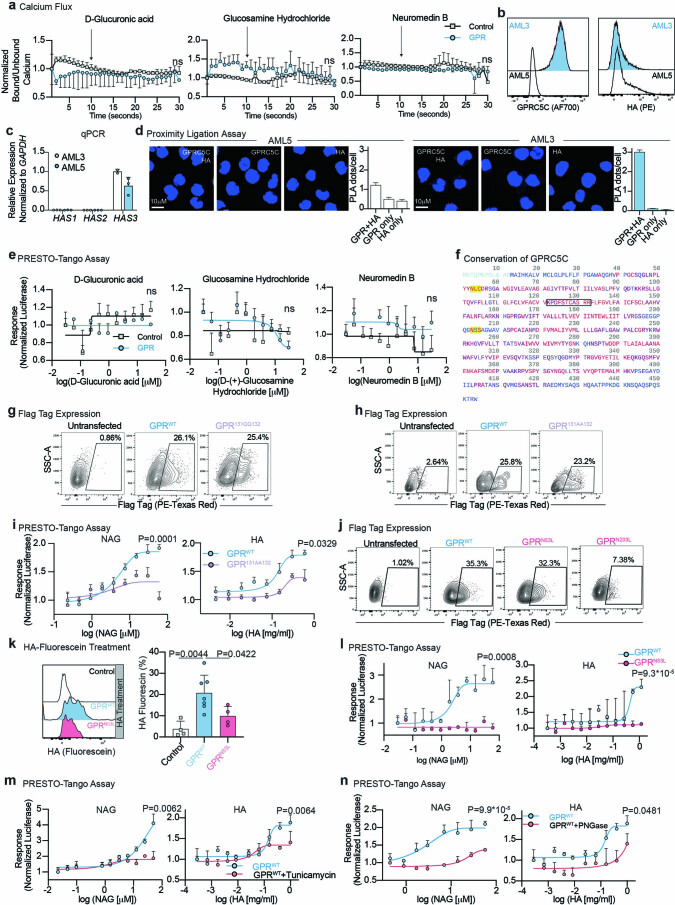
**a**. Normalized ratio of baseline calcium level measured by Indo-1 followed by calcium flux in response to D-glucuronic acid (GA), glucosamine hydrochloride (G), and neuromedin B (NMB) in GPRC5C-OE (*n* = 3 experiments) and control (*n* = 4 experiments) in AML3 cells. **b**. HA and GPRC5C expression in AML3 and AML5. **c**. *HAS1,2*, and *3* expression in AML3 and AML5. *n* = 3 experiments, except for *HAS3* expression in AML3 *n* = 2 experiments. **d**. Technical PLA controls of GPRC5C and HA in AML3 and AML5. Plot is representative of one out of three independent experiments. Each independent experiment was performed in two technical replicates. *n* = 543, 551, 463, 990, 503, and 523 cells for AML3: GPR + HA, GPR only, HA only and AML5: GPR + HA, GPR only, and HA only, respectively. Data presented as mean ± SEM. **e**. Concentration–response curve of PRESTO-Tango construct of GPRC5C and control vector treated with GA, G, and NMB in HTLA cells. Normalized to luciferase response at the lowest concentration. *n* = 4 experiments. **f**. Residue conservation of GPRC5C based on a multiple sequence alignment of 250 homologous proteins extracted from UniRef90. Red: highly conserved; purple: conserved; blue: not conserved; light blue: no conservation information available; yellow background: sequon for N-glycosylation used for mutation analysis within the ECD of the protein; black box: the putative BX_7_B domain. **g**. FACS validation of surface protein levels of GPRC5C^WT^ and site-specific mutant (GPRC5C^131QQ132^) in HTLA cells. **h**. FACS validation of surface protein levels of GPRC5C^WT^ and site-specific mutant (GPRC5C^131AA132^) in HTLA cells. **i**. Concentration-response curve of PRESTO-Tango construct for GPRC5C^WT^ and site-specific mutant (GPRC5C^131AA132^) at predicted HA interaction site in HTLA cells. *n* = 6 experiments. **j**. FACS validation of surface protein levels of GPRC5C^WT^ and site-specific mutants (GPRC5C^N53L^ and GPRC5C^N203L^) in HTLA cells. **k**. HA-fluorescein-treated cells quantified for GFP retention in HEK293T cells expressing GPRC5C^WT^ and GPRC5C^N53L^. FACS plots representing HA retention in each condition. *n* = 4 experiments, except for GPRC5C^WT^ where *n* = 7 experiments. **l**. Concentration-response curve of PRESTO-Tango construct for GPRC5C^WT^ and site-specific mutant (GPRC5C^N53L^) at predicted HA interaction site in HTLA cells. *n* = 6 experiments. **m**. Concentration-response curve of PRESTO-Tango construct for GPRC5C^WT^ treated with tunicamycin in HTLA cells. *n* = 3 experiments. **n**. Concentration-response curve of PRESTO-Tango construct for GPRC5C^WT^ treated with PNGase in HTLA cells. *n* = 3 experiments. All data presented as mean ± SD, except where otherwise stated. Statistical significance was determined using two-tailed t-test. ns, not significant. *n* indicates the number of replicates. For all experiments, at least two independent experiments were performed. Source numerical data are available in source data. Source data

GPRC5C is a seven-transmembrane orphan receptor^27^. To predict potential regulators of dormancy, we performed in silico structural modelling of GPRC5C. Briefly, the full length of GPRC5C isoform 2 was modelled using a recombinant approach of homology and *ab initio* modelling (Fig. 5a and Methods). Like other members of the G-protein-coupled receptor (GPCR) superfamily, three distinct regions were identified in GPRC5C, namely: an extracellular domain (ECD), a seven-transmembrane domain (7TM), and an intracellular domain. However, unlike all other class C GPCRs, the ECD of GPRC5C lacks a characteristic venus fly trap (VFT) domain, which is the orthosteric binding site^28–30^. Instead, the ECD of GPRC5C consists of three α-helices and two β-helices, along with the loop regions that connect the ECD to the 7TM, revealing a topology more closely related to class A than class C GPCRs. In silico molecular docking suggested that HA may interact with GPRC5C through the 7TM (Fig. 5a). Interestingly, HA activates class A GPCR κ-opioid receptors, although the mechanism of action is not yet understood^31^. HA is a major component of the BM niche and facilitates homing and binding of HSCs to endothelial cells^32,33^. In GPRC5C-OE cells, we found a significant increase in expression of GNA11 and GNA14, which are part of the G_q_ alpha subunit (Extended Data Fig. 6s). Activation of G_q_-coupled GPCRs leads to a transient increase in intracellular calcium concentrations^34^. To measure calcium concentrations upon potential agonist interaction, we overexpressed GPRC5C and the respective empty vector control in AML3 cells and loaded cells with Indo-1, a fluorescent Ca^2+^ indicator dye (Fig. 5b). We tested HA and its subunits *N*-acetyl-d-glucosamine (NAG) and glucuronic acid (GA) to elicit a spike in calcium. Addition of HA and NAG induced an increase in intracellular calcium levels, indicating that both activate GPRC5C signalling (Fig. 5b). In contrast, treatment with GA showed no changes in intracellular calcium (Extended Data Fig. 7a). As additional negative controls, we used glucosamine hydrochloride (a form of NAG lacking the amide group) and neuromedin B (NMB) (a known ligand of the NMB receptor), which we did not expect to interact with GPRC5C. As predicted, treatment with glucosamine hydrochloride and NMB did not alter intracellular calcium (Extended Data Fig. 7a).

To further support the interaction between HA and GPRC5C, we performed a proximity ligation assay (PLA; Fig. 5c). We used the AML3 and AML5 cell lines, since we found that both produce endogenous HA (due to expression of the HA-producing enzyme *HAS3*) but only AML3 cells express GPRC5C (Extended Data Fig. 7b,c), avoiding the genetic modification of cells. We observed a significantly higher number of fluorescent dots in AML3 versus AML5 cells, indicating a proximity of less than 80 nm between GPRC5C and HA (Fig. 5c and Extended Data Fig. 7d). Next, we overexpressed GPRC5C and treated cells with an HA-conjugated fluorescein. GPRC5C-OE cells showed higher HA interaction compared with the control condition (Fig. 5d). To confirm NAG as the interacting subunit of HA, we designed a competitive interaction experiment. GPRC5C-OE cells were simultaneously treated with HA-conjugated fluorescein and non-fluorescent NAG. HA interaction with GPRC5C was reduced when NAG was present, suggesting that HA competes with NAG (Fig. 5d).

The desensitization of GPCRs depends on the recruitment of β-arrestin to the receptor, which uncouples the receptor from the G protein and promotes internalization and deactivation^35^. In the PRESTO-Tango assay, β-arrestin recruitment is quantified through luciferase^36^ (Fig. 5e). We treated GPRC5C-OE HTLA cells with varying concentrations of HA, NAG and negative control compounds such as GA, glucosamine hydrochloride and NMB. Quantification of luciferase revealed that HA and NAG treatments led to a dose-dependent response (Fig. 5e), while treatment with GA, NMB and glucosamine hydrochloride did not elicit a luciferase response (Extended Data Fig. 7e). Together, these results suggest that HA and NAG induce GPRC5C activation.

To further dissect the underlying mechanism of GPRC5C activation by HA, we examined the putative mode of interaction. For some HA-binding proteins, a B(X_7_)B motif was described, where ‘B’ represents R (arginine) or K (lysine), and ‘X’ represents any non-acidic amino acid and at least one basic amino acid^37–40^. The 122KPDFSTCASRR132 motif connects TM2 and TM3 in GPRC5C. This motif is highly conserved between species and most closely resembles the B(X_7_)B motif (Extended Data Fig. 7f, black box). Further examination of contacting residues by molecular docking revealed that the interactions could be mediated through a strong network of hydrogen bonds with K122, along with a salt-bridge interaction with R132. To experimentally analyse the putative HA-binding site, we mutated 131RR132, two basic amino acids within the proposed B(X_7_)B domain, to 131QQ132 (Extended Data Fig. 7f). We transfected GPRC5C^WT^ and GPRC5C^131QQ132^ constructs and observed similar protein levels, as indicated by the Flag epitope conjugated at the N-terminus of GPRC5C (Extended Data Fig. 7g). GPRC5C^131QQ132^ mutation led to a reduction in the ability to retain HA-conjugated fluorescein, and a reduced luciferase response compared with GPRC5C^WT^ cells, further supporting the putative HA–GPRC5C interaction site (Fig. 5f,g). These results were additionally validated using an 131RR132 to 131AA132 GPRC5C mutant, which mimicked the loss of function previously observed in the GPRC5C^131QQ132^ mutant (Extended Data Fig. 7h,i).

In eukaryotic cells, NAG is attached to an asparagine (N) as part of a process known as *N*-glycosylation. In many cases, *N*-glycosylation is critical for the structure and function of the protein and can modulate the ligand on-and-off rate^41^. The classical sequon for *N*-glycosylation is N-X-S/T (X ≠ P). This sequon was located at N^203^-S^204^-S^205^ in GPRC5C. An additional putative C-containing glycosylation motif (N-X-C) was located at N^53^-K^54^-C^55^. This rare motif for the attachment of *N*-linked glycans is essential in some proteins^42^. To test whether glycosylation of these sites may be important for HA and NAG interaction, we generated GPRC5C^N53L^ and GPRC5C^N203L^ mutants. Using Flag-tag expression, we confirmed the presence of GPRC5C^N53L^ on the cell membrane, while the GPRC5C^N203L^ mutant was reduced on the cell surface (Extended Data Fig. 7j). Using HA-conjugated fluorescein, we showed that the GPRC5C^N53L^ mutation reduced GPRC5C’s ability to retain HA and dampened the luciferase response that was previously elicited by treatment with HA and NAG (Extended Data Fig. 7l,k). Similarly, *N*-glycosylation inhibitors such as PNGase and tunicamycin^43,44^ hindered the ability of HA and NAG to elicit a luciferase response (Extended Data Fig. 7m,n). This suggests that NAG may partly modulate the activity of GPRC5C through the *N*-glycosylation of GPRC5C, which is critical for GPRC5C activation.

In summary, these data show that HA and its subunit NAG are regulators of GPRC5C activation.

### HA–Gprc5c signalling axis regulates HSCs

**Fig. 6:**
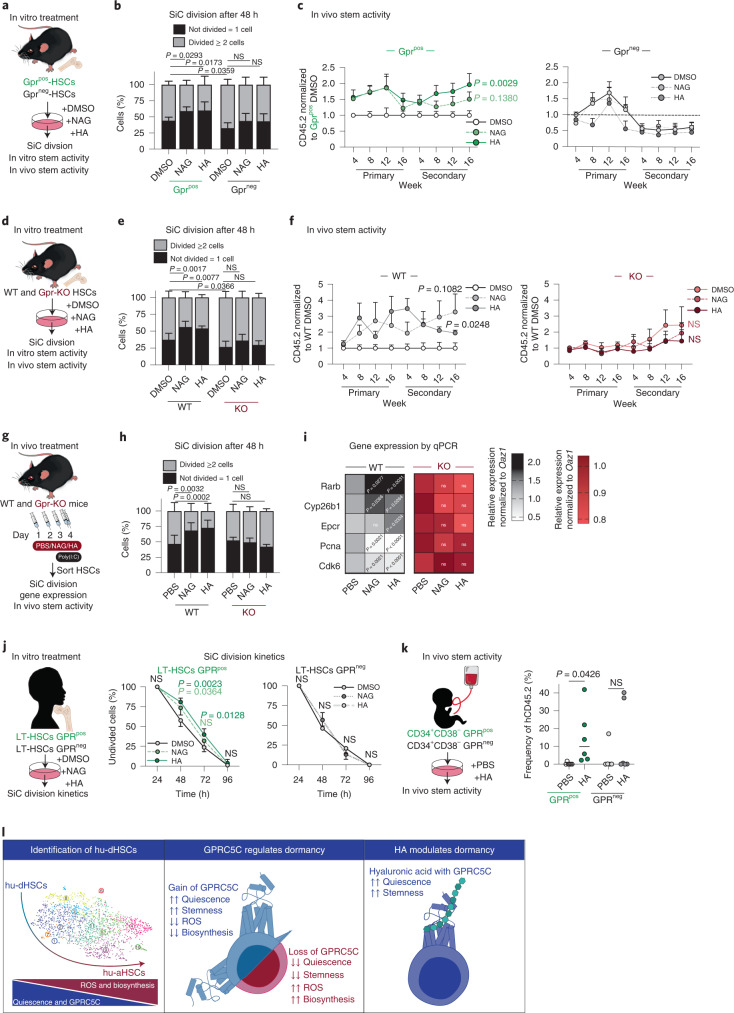
HA–Gprc5c signalling axis regulates human and mouse HSCs. **a**, Experimental design to assess the effect of in vitro HA and NAG treatment on Gprc5c^pos/neg^–HSCs. **b**, SiC division assay of Gprc5c^pos/neg^–HSCs quantified after 48 h in vitro culture treatment with NAG, HA and DMSO control. *n* = 8 biological replicates. **c**, Transplantation of Gprc5c^pos/neg^–HSCs treated with NAG, HA and DMSO control. *n* = 7 biological replicates, except *n* = 5 biological replicates for Gprc5c^pos^-HSCs treated with NAG. **d**, Experimental design to assess the effect of in vitro HA and NAG treatment on Gprc5c-KO and WT control HSCs. **e**, SiC division assay of Gprc5c-KO and WT control HSCs quantified after 48 h in vitro culture treatment with NAG, HA and DMSO control. *n* = 6 biological replicates. **f**, Transplantation of Gprc5c-KO and WT control HSCs treated with NAG, HA and DMSO control. *n* = 9, 9, 6, 11, 12 and 17 biological replicates for WT-DMSO, WT-NAG, WT-HA, Gpr-KO-DMSO, Gpr-KO-NAG and Gpr-KO-HA, respectively. **g**, Experimental design to assess the effect of in vivo NAG and HA treatment in preventing viral stress from poly I:C. HSCs defined as Lineage^−^cKit^+^Epcr^+^CD150^+^CD48^−^CD34^−^. **h**, SiC division assay of in vivo treated Gprc5c-KO and WT control HSCs cells quantified after 48 h of in vitro culture. *n* = 6 biological replicates. **i**, Differential gene expression. Heat map representing median RNA expression from qPCR data (normalized to housekeeping gene Oaz1 and PBS control) *n* = 11 biological replicates. **j**, SiC division kinetics of BM GPRC5C^pos/neg^–LT-HSCs with in vitro culture treatment with NAG, HA and DMSO control. *n* = 4 biological replicates. **k**, Transplantation of GPRC5C^pos/neg^-HSCs from two CB donors treated with HA and control. *n* = 6 biological replicates. **l**, Representative summary of findings. All data presented as mean ± s.d., except **i** where median is presented. Statistical significance was determined using two-tailed *t*-test (**c**, **f**, **i** and **k**) or two-way ANOVA (**b**, **e**, **h** and **j**). *n* indicates number of biological replicates. For all experiments, at least two independent experiments were performed. Source numerical data are available in source data. Source data

**Extended Data Fig. 8:**
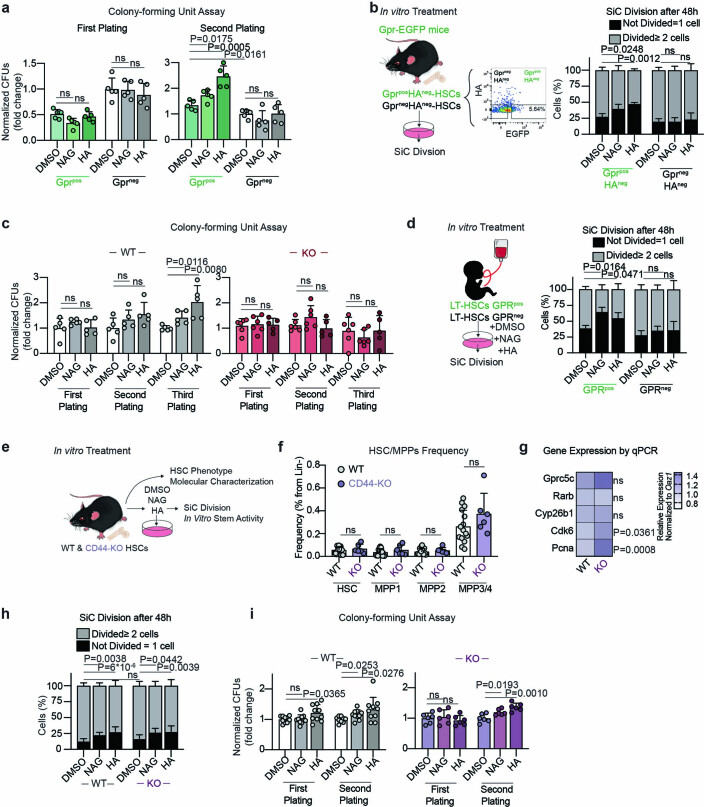
**a**. Serial CFU assay of Gprc5c^pos/neg^–HSCs cultured in NAG, HA, or DMSO control for 72 hours. *n* = 6 biological replicates. **b**. Left panel: experimental design to assess the effect of *in vitro* HA, NAG, and DMSO treatment on HA^neg^Gprc5c^pos/neg^–HSCs. Right Panel: SiC division assay of HA^neg^Gprc5c^pos/neg^–HSCs quantified after 48 hours *in vitro* culture treatment with NAG, HA, and DMSO. *n* = 5 biological replicates. **c**. Serial CFU assay of Gprc5c-KO and WT control HSCs cultured in NAG, HA, and DMSO control for 72 hours. *n* = 5, 3, 5, 6, 6, and 5 biological replicates for WT-DMSO, WT-NAG, WT-HA, Gpr-KO-DMSO, Gpr-KO-NAG, and Gpr-KO-HA, respectively. **d**. SiC division assay of GPRC5C^pos/neg^–LT-HSCs with *in vitro* culture treatment for 48 hours with NAG, HA, and DMSO control. *n* = 4 biological replicates. **e**. Experimental design to assess the effect of *in vitro* NAG, HA, and DMSO treatment on CD44-KO and WT control mice. **f**. Frequency of HSC/MPP populations from 8^−^week-old CD44-KO (*n* = 6 biological replicates) and WT control (*n* = 12 biological replicates) mice. **g**. Differential gene expression. Heatmap representing median RNA expression from qPCR data (normalized to housekeeping gene *Oaz1* and WT control). *n* = 7 biological replicates. **h**. SiC division assay of CD44-KO and WT control HSCs quantified after 48 hours *in vitro* culture treatment with NAG, HA and DMSO. *n* = 15, 15, 13, 8, and 12 biological replicates for WT-DMSO, WT-NAG, WT-HA, CD44-KO-DMSO, CD44-KO-NAG, and CD44-KO-HA, respectively. **i**. Serial CFU assay of CD44-KO (*n* = 6 biological replicates) and WT control (*n* = 10 biological replicates) HSCs cultured *in vitro* with NAG, HA, and DMSO for 48 hours. All data presented by mean ± SD. Statistical significance was determined using two-tailed *t-test* (a, c, f, g, i) or two-way ANOVA (b,d, h). ns, not significant. *n* indicates number of biological replicates. For all experiments, at least two independent experiments were performed. Source numerical data are available in source data. Source data

To test whether the presence of HA and NAG could maintain quiescence and stemness, we sorted mouse Gprc5c^pos/neg^-HSCs on the basis of enhanced green fluorescent protein expression (EGFP) and treated them with HA or NAG for in vitro single-cell division, CFU assay, and in vivo transplantations (Fig. 6a). We observed that HA and NAG treatment induced quiescence, and enhanced in vitro and in vivo self-renewal capacity, in Gprc5c^pos^-HSCs specifically (Fig. 6b,c and Extended Data Fig. 8a). To exclude potential bias from HA^pos^ cells, we sorted Gprc5c^pos/neg^-HSCs, which were HA^neg^ and treated with HA and NAG; we nevertheless observed delayed division within the Gprc5c^pos^-HSCs specifically (Extended Data Fig. 8b).

Gprc5c-KO and WT HSCs were used to assess whether HA and NAG regulate HSCs through Gprc5c signalling (Fig. 6d). Treatment of WT HSCs with HA and NAG resulted in delayed division, increased CFU capability upon serial plating and enhanced in vivo reconstitution capacities upon transplantation (Fig. 6e,f and Extended Data Fig. 8c). However, in vitro HA and NAG treatment of Gprc5c-KO HSCs did not affect quiescence, in vitro self-renewal capacity or in vivo self-renewal capacity compared with WT HSCs (Fig. 6e,f and Extended Data Fig. 8c).

Next, we performed serial intraperitoneal injection of HA and NAG followed by poly I:C injection, which is known to drive HSCs into proliferation^45^ (Fig. 6g). In vitro single-cell division analysis revealed that HSCs from WT mice treated with HA and NAG had delayed division compared with the control condition (Fig. 6h). Genes associated with HSC dormancy (Rarb and Epcr) were upregulated and genes associated with cell cycle (Pcna and Cdk6) were downregulated in HA- and NAG-treated mice (Fig. 6i). In contrast, we did not observe any changes in the division or gene expression of HSCs when Gprc5c-KO mice were subjected to the same conditions (Fig. 6h,i). These results suggest that HA and NAG treatment preserves mouse HSC quiescence via Gprc5c.

To test the conservation of HA-regulated quiescence in humans, we monitored the division kinetics of HA- and NAG-treated GPRC5C^pos/neg^-LT-HSCs from BM and CB. We found that treatment with HA and NAG resulted in delayed division in GPRC5C^pos^-LT-HSCs specifically (Fig. 6j and Extended Data Fig. 7d). Higher engraftment potential was observed in HA-treated GPRC5C^pos^-HSCs. Conversely, HA treatment had a limited effect on self-renewal capabilities in GPRC5C^neg^-HSCs (Fig. 6k).

CD44 is the most well-described receptor for HA and has been shown to be critical in supporting mouse stem and progenitor cell homing to the BM niche^46,47^. To dismiss the possibility that HA-mediated quiescence and self-renewal is established via the CD44–HA signalling axis, we examined HA and NAG treatment in mouse CD44-KO HSCs (Extended Data Fig. 8e). Phenotypic characterization of CD44-KO BM cells revealed no differences in the frequency of HSCs or MPPs (Extended Data Fig. 8f). CD44-KO HSCs showed increased expression of cell cycle-associated genes (Cdk6 and Pcna) but no alteration in expression of dormancy-associated genes (Gprc5c, Rarb and Cyp26b1) (Extended Data Fig. 8g). HSCs treated with NAG/HA had delayed primary division and showed enhanced in vitro self-renewal capacity irrespective of whether they were WT or CD44-KO HSCs (Extended Data Fig. 8h,i). These results are consistent with a previous report showing that deletion of CD44 in adult BM HSCs has no impact on long-term reconstitution^48^, suggesting an alternative mechanism. Additionally, HA interacts with other receptors such as LYVE-1, TLR4 and RHAMM^37,49,50^. Thus, securing definite evidence of the HA–GPRC5C interaction will only be possible by solving the crystal structure of GPRC5C.

Together, these results suggest that HSC quiescence is regulated by HA through its interaction with GPRC5C.

## Discussion

In this study, we found that human BM HSCs are organized on an axis of dormancy (Fig. 6l). This dormancy axis is characterized by gradual upregulation of processes such as translation, ribosomal biosynthesis, cell cycle and ROS. hu-dHSCs resembled mouse dHSCs, previously described only within the murine haematopoietic system, and quiescent populations within human CB^5,10–13^. We found that the orphan receptor GPRC5C enriches for HSC dormancy and plays an important role in regulating mouse and human HSC features. GPRC5C^pos^ HSCs are highly quiescent, show high in vivo reconstitution capacities, and are regulated by the extracellular matrix molecule HA (Fig. 6l).

Class C GPCRs are defined by two unique structural features: first, class C GPCRs contain a large ECD comprising a VFT and a cysteine-rich domain; second, they form constitutive dimers through the VFT^29^. GPRC5C is unique because its protein sequence is much shorter compared with other members of class C. Our in silico modelling revealed that the region of GPRC5C spanning the extracellular matrix contains 90 amino acids, making it structurally difficult to form the VFT module (typically between 500 and 600 amino acids). GPRC5C, along with GPRC5A and GPRC5B, is classified as a class C GPCR owing to sequence homology. However, its topology has revealed that it may be more closely related to class A GPCRs^51^. Given the distinctive differences revealed by the in silico model of GPRC5C compared with other members of class C GPCRs, its reclassification or subclassification may be warranted. Using in silico modelling and mutational cell-based assays, we have proposed a potential model of HA interaction with the 7TM domain of GPRC5C. However, it remains unclear if a mutation in the HA-binding domain of GPRC5C would result in loss of dormancy in human HSCs. Investigation of the functionality of these mutations would require CRISPR–Cas9 technologies, which are still challenging to use in human LT-HSCs owing to the limited effectiveness of long-term cultivation of human HSCs.

Some uncertainty remains regarding the proposed binding mode of HA since the in silico modelling is dependent on existing structures; however, there are few experimentally determined atomic models of the GPCR class C family. To address the precise mechanism by which GPRC5C binds to HA and NAG would require further biophysical assays, such as co-crystallization or cryo-electron microscopy. However, isolation and crystallization of membrane proteins has proven notoriously difficult. Further, GPCRs are often purified in the presence of their orthosteric or allosteric ligands, particularly antagonists and inverse agonists, to maintain their conformationally stabilized form^52^. The orthosteric ligand for GPRC5C is not yet known, and identification would require intensive high-throughput screening. Furthermore, we cannot discount the possibility that other known HA receptors, such as TLR4, may also play a role in regulating dormancy. Nonetheless, our extensive cell-based assays show that GPRC5C is activated by NAG and HA, thus allowing us to identify a signalling axis that regulates HSC dormancy.

In summary, we have found that maintaining a deep state of quiescence, or dormancy, is regulated by the GPRC5C–HA signalling axis in both mouse and human HSCs.

## Methods

### Mouse models

All mice were bred in-house in the animal facility at the Max Planck Institute of Immunobiology and Epigenetics in individually ventilated cages. Mice were killed by cervical dislocation according to German guidelines. Animal procedures were performed according to the protocols approved by the German authorities, Regierungspräsidium Freiburg (the killing of animals for scientific purposes according to §4 (3) of the German Animal Protection Act); animal protocol numbers: G-18/142, G-19/74 and G-20/124.

### Isolation of mouse HSCs

Murine BM cells were isolated from the femur, tibia, hip bone and vertebrae by gentle crushing with mortar and pestle in PBS. Red blood cell lysis was performed with ACK Lysing Buffer (Thermo Fisher Scientific) for 5 min at room temperature (RT). Dynabeads Untouched Mouse CD4 Cells Kit (Invitrogen) was used for lineage negative enrichment according to the manufacturer’s protocol. Briefly, the BM was stained with 1:4 dilution of the Lineage Cocktail for 30–60 min at 4 °C on a rotating wheel. Labelled cells were then incubated for 20 min with 400 µl of washed Dynabeads coated with polyclonal sheep anti-rat IgG per sample. Depletion of lineage cells was performed using a magnet. Lineage-depleted BM cells were stained with lineage markers (Gr1, CD11b, B220, Ter119, CD4 and CD8a), ckit, Sca1, CD150, CD48 and CD34 and sorted on an Aria Fusion II for downstream analysis.

### Mouse transplantation

#### HSC transplants

In total, 100 HSCs (LSK CD150^+^CD48^−^CD34^−^) from Gprc5c KO and WT controls were sorted and transplanted by tail-vein injection into fully irradiated CD45.1 recipients (4.5 + 5 Gy).

#### Competitive WBM chimaeras

Two sets of chimaeras were generated: CD45.1/.2 WT + CD45.2 WT and CD45.1/.2 WT + CD45.2 Gprc5c KO. In every case, 10^6^ and 10^6^ WBM cells were mixed. Mixed WBM were transplanted into recipients as described above.

#### Reverse transplantation

A total of 10^6^ WBM from WT CD45.1 mice were isolated and transplanted into recipients, either Gprc5c-KO or littermate controls, as described above.

#### Secondary transplants

WBM cells were isolated after 16 weeks post-transplantation, and 10^6^ WBM cells were re-transplanted into fully irradiated CD45.1 recipients.

For all transplanted mice, the contribution of donor cells was monitored in PB obtained from the submandibular vein at 4, 8, 12 and 16 weeks post-transplantation.

### Homing

For analysis of homing capacity, 5 × 10^6^ WBM cells (WT or Gprc5c-KO CD45.2) were transplanted into fully irradiated CD45.1 recipients (4.5 + 5 Gy) and killed 48 h later. CD45.1/2 staining was performed, and engraftment was analysed by flow cytometry analysis.

### 5-FU injections

Once a week, WT and Gprc5c-KO mice were intraperitoneally injected with 150 mg kg^−1^ 5-FU (Sigma) dissolved in PBS, with a final concentration of 20 mg ml^−1^. Body weight was checked weekly. Mice were killed when 20% of their initial weight was lost.

### NAG and HA in vivo treatments

WT and Gprc5c-KO mice were intraperitoneally injected with either PBS, 100 mg kg^−1^ NAG or 60 mg kg^−1^ HA for four consecutive days. Additionally, 50 µg of polyI:polyC (poly I:C) was injected intraperitoneally on day 4. On day 5, mice were killed and BM cells were isolated for further analysis.

### NBSGW transplantations

#### Endogenous GPRC5C

In total, 7,000 BM or 800 CB GPRC5C^pos/neg^-CD34^+^CD38^−^ cells were sorted into PBS. Before transplantation, the number of cells was validated by haemocytometer and then injected intravenously into 7- to 10-week-old female NBSGW mice that were irradiated with 1.5 Gy. Engraftment was monitored by PB obtained from the submandibular vein. After 24 or 36 weeks, mice were killed and BMs (hip bone, tibia and femur) were analysed for human chimaerism.

#### GPRC5C-OE

A total of 10^4^ CD34^+^ CB cells were transduced after 24 h of pre-stimulation. The transduction efficiency was assessed by flow cytometry after 2 days of cultivation. Total culture was injected intravenously into 7- to 10-week-old female NBSGW mice as stated above. After 24 weeks, mice were killed and BM was analysed for human chimaerism along with GFP. Primary engraftment was calculated by total hCD45^+^GFP^+^ engraftment normalized to the initial transduction efficiency of GFP.

For purification of human cells from xenotransplanted mice, BM from individual mice was enriched for human cells with the Mouse Cell Depletion Kit (Miltenyi Biotec) according to the manufacturer’s protocol, and 140,000 GFP^+^ cells were sorted and re-transplanted. A mouse was considered engrafted if the percentage of hCD45^+^ was greater than or equal to 0.1%.

### Cell cycle analysis (Ki-67 or CDK6)

Stained cells were fixed with BD Cytofix/Cytoperm Buffer (BD Biosciences) for 10 min at 4 °C. Next, cells were stained with intracellular Ki-67 at 1:100 (BD Biosciences) or CDK6 at 1:500 (Abcam) in PermWash solution (BD Biosciences) for 45–90 min at 4 °C. Before cell cycle analysis on the BD LSRFortessa Cell Analyzer (BD Biosciences) using BD FACSDiva v8.0.3, cells were stained in the dark with Hoechst 33342/DAPI (Invitrogen) for 30 min at RT.

### Immunofluorescence quantification of CDK6

GPRC5C^pos/neg^-CD34^+^CD38^−^ were sorted by the FACSAria Fusion II flow cytometer (BD Biosciences) directly on polylysine-coated slides and were fixed over 10 min with BD Cytofix/Cytoperm Buffer (BD Biosciences) at RT. Fixed cells were permeabilized with 0.2% Triton (Sigma) for 10 min and blocked for 20 min with 10% goat serum (Life Technologies). Cells were stained with 1:500 of CDK6 Alexa Fluor 647(Abcam) for 1 h and washed with PBS. Slides were visualized on the LSM 780 confocal microscope (Zeiss) using ZEN blue v2.5, and fluorescence quantification was performed with ImageJ.

### CellROX/MitoTracker staining

Cells were incubated at 37 °C with CellROX DeepRed at 1/500 (Invitrogen) or MitoTracker Red at 50 nM (Invitrogen) in their corresponding media for 30 min. Cells were subsequently washed three times in PBS and stained for FACS analysis on the BD LSRFortessa Cell Analyzer (BD Biosciences).

### *O*-propargyl-puromycin

Stained cells were fixed for 15 min at RT with BD Cytofix/Cytoperm Buffer (BD Biosciences) and permeabilized with 0.1% Triton for 15 min at RT. The copper-catalysed azide-alkyne cycloaddition was performed using an Alexa594-azide (Life Technologies, 5 mM final concentration) and the Click-iT Cell Reaction Buffer Kit (Life Technologies) according to the manufacturer’s instructions.

### Single-cell differentiation assay

Anonymized BM CD34^+^ samples were purchased from Lonza Group Ltd with informed consent from healthy donors. BM samples were stored in accordance with regulated procedures and the Human Tissue Act, and with protocols approved by the relevant research and ethics committees (covered in Cambridge by the Cambridge Blood and Stem Cell Biobank, IRAS ref. 149581).

To isolate cell populations from BM CD34^+^ cells, frozen samples were thawed by drop-wise addition of pre-warmed Iscove’s modified Dulbecco’s medium (IMDM; Life Technologies) + 0.1 mg ml^−1^ DNase (Sigma) + 50% foetal bovine serum (FBS, Life Technologies). Cells were counted before re-suspension in an antibody mix with a panel of cell-surface markers (5 × 10^6^ cells ml^−1^ in Stemspan antibody mix) (CD34, CD38, CD45RA, CD90, CD49f, Zombie, CD19, biotinylated hyaluronan and streptavidin) for 30 min at 4 °C, followed by washing with 1 ml of 3% PBS/FCS. BM LT-HSCs were sorted using single-cell purity and index sorting to allow retrospective correlation between cell-surface marker expression from each single cell with in vitro functional lineage output. GPRC5C^pos/neg^ BM LT-HSCs were determined retrospectively by index analysis after single-cell sorting.

MS5 stromal cells were imported from Prof. Katsuhiko Itoh at Kyoto University and were tested to support myeloid (My), erythroid (Ery), megakaryocytic (Meg) and natural killer (NK) differentiation with appropriate control populations. MS5 cells were plated at passage 10–13 at 3,000 cells per well in flat 96-well plates in 100 μl Myelocult H5100 medium (StemCell Technologies) + 1% Pen/Strep (Life technologies) 1 day before sorting BM LT-HSCs. On the day of the sort, the medium was changed to 100 μl per well MEM cytokine medium to support the growth of My, Ery, Meg and NK colonies. MEM medium composition included StemPro base medium supplemented with nutrients (0.035%) (Life Technologies) and the following cytokines: SCF 100 ng ml^−1^, Flt3-L 20 ng ml^−1^, TPO 100 ng ml^−1^, IL-6 50 ng ml^−1^, IL-3 10 ng ml^−1^, IL-11 50 ng ml^−1^, GM-CSF 20 ng ml^−1^, IL-2 10 ng ml^−1^ and IL-7 20 ng ml^−1^ (all Miltenyi Biotech); erythropoietin (EPO) 3 units ml^−1^ (Eprex, Janssen-Cilag); h-LDL 50 ng ml^−1^ (StemCell Technologies); 1% l-glutamine (Life Technologies); and 1% penicillin–streptomycin (Life Technologies). Indicated populations were index-sorted as single cells (one cell per well) and cultured for 3 weeks at 37 °C and 5% CO_2_.

### PLA

Each sample of 80,000 cells was rested on diagnostic microscope slides (Thermo Fisher Scientific) at 37 °C for 45 min. Cells were fixed with 4% paraformaldehyde for 20 min at RT and blocked for 1 h. Blocked cells were stained with the Duolink kit (Sigma Aldrich) according to the manufacturer’s instructions. The antibody combination used was mouse anti-GPRC5C (R&D systems) and rabbit anti-HA (LS Bio). Nuclei were stained with DAPI (Roth). Images were taken at 60× magnification with a confocal microscope (Nikon C2) and analysed with BlobFinder.

### Single-cell differentiation assay analysis

All single-cell-derived colonies were collected into 96 U-bottom plates using a plate filter to prevent carryover of MS5 cells. Cells were then stained with 50 μl per well of antibody mix (CD66, CD15, CD14, CD56, CD71, CD45, GlyA, Cd41a and CD11b), incubated for 20 min in the dark at RT, and then washed with 3% PBS/FBS (100 μl per well). The lineage composition and the size of colonies formed was assessed by high-throughput flow cytometry. A single cell was defined as giving rise to a true colony if the sum of cells detected in the CD45^+^ and GlyA^+^ gates was ≥30 cells and the colony output was determined. Ery colonies were identified as CD45^−^GlyA^+^ ≥30 cells, Meg colonies as CD45^−^CD41^+^ CD15^−^CD14^−^ ≥20 cells, My colonies as [(CD45^+^CD14^+^) + (CD45^+^CD15^+^)] ≥30 cells and NK colonies as CD45^+^CD56^+^ ≥30 cells. If a true colony was found but did not meet any threshold for lineage determination, the colony was designated as ‘undifferentiated’. Colony analysis was performed in RStudio v1.25.

### Single-cell division

Single cells were FACS-sorted into individual wells on 72-well Terasaki plates (Greiner Bio-One) with 10 µl of StemPro or Stemspan with complete cytokines. Cells were evaluated for division under a light microscope 48 or 72 h after sorting.

### Primary cell culture

Human CD34^+^ cells were cultured in HSC medium, consisting of StemSpan ACF (StemCell Technologies) supplemented with 100 ng ml^−1^ human stem cell factor (PeproTech), 100 ng ml^−1^ FMS-like tyrosine kinase 3 ligand (PeproTech), 50 ng ml^−1^ thrombopoietin (PeproTech), 10 μg ml^−1^ human low-density lipoprotein (StemCell Technologies) and 1× Glutamax (Thermo Fisher Scientific) with or without UM171 at 35 nM.

Primary murine haematopoietic cells were cultured in StemPro-34 SFM (Life Technologies) with supplement and 50 ng ml^−1^ SCF, 25 ng ml^−1^ TPO, 30 ng ml^−1^ Flt3-Ligand (all PeproTech), 100 U ml^−1^ penicillin–streptomycin and 2 mM l-glutamine (Thermo Fisher).

### CFU assays

#### Mouse CFU

In total, 200–400 HSCs were either FACS-sorted directly into 1 ml of MethoCult M3434 (StemCell Technologies) or into 384-well plates containing 100 µl Complete Stem Cell Media was supplemented with HA (250–500 µg ml^−1^), NAG (20 µM) or DMSO control. After 48–72 h of in vitro treatment, total cultured HSCs were collected into 1 ml of MethoCult M3434 (StemCell Technologies). Approximately 5 days after primary plating, the number of colonies was quantified. Then, 10^4^ cells were used for secondary, tertiary and quaternary plating. Approximately 5 days after each replating, colonies were quantified and replated.

#### Human CFU

A total of 10^3^ GFP^+^ cells were sorted into 1 ml of MethoCult H4435 (StemCell Technologies). Seven days after primary plating, the colony numbers were quantified. After 14 days, 10^4^ cells were replated and quantified.

### HA–fluorescein binding assay

HEK293T cells were transfected with 30 μg ml^−1^ of the GPRC5C-Tango plasmids using JetPrime transfection reagent. After 24–36 h transfection, 60,000–80,000 cells were treated with 8 μg HA conjugated to fluorescein with or without 100 µM NAG for 60 min at RT with shaking. Cells were washed, and fluorescein was quantified using the LSR II flow cytometer (BD Bioscience).

### qRT–PCR analysis

For real-time quantitative PCR (qPCR), the total RNA of 1,000–2,000 cells was isolated with the Arcturus PicoPure RNA isolation kit (Applied Biosystems) according to the manufacturer’s guidelines. First-strand synthesis was reverse transcribed using the SuperScript VILO cDNA Synthesis Kit (Invitrogen) according to the manufacturer’s guidelines. For qPCR analysis, Fast SYBR Green Master Mix or TaqMan probes (Thermo Fisher) with TaqMan Fast Advanced Master Mix (Thermo Fisher) were used on a StepOne Real-Time PCR System (Applied Biosystems) or QuantStudio Flex 6 Real-Time PCR System (Applied Biosystems). RNA expression was normalized to expression of *Oaz1* or *GAPDH* housekeeping genes and presented as relative quantification (ratio 2^−ΔΔCT^).

### Virus production and primary cell infection

Lentiviral particles were produced in HEK293T cells. In each 150 mm tissue culture dish, 15 μg of the lentiviral vector was transiently packaged with 9 μg psPAX2 and 6 μg pMD2.G with JetPrime (Polyplus). Viral supernatant was collected at 48 h post-transfection. Virus concentration was achieved by ultracentrifugation at 30,000 rpm through a 20% sucrose cushion for 120 min at 4 °C. For infection of CD34^+^ cells, each well of non-tissue-culture-treated 96-well plates (Corning) was coated with 6.4 μg RetroNectin reagent (Takara Clontech) in 100 μl PBS and incubated overnight at 4 °C. The coated wells were washed and blocked with PBS, containing 2% BSA, for 30 min at RT. Viral particles (multiplicity of infection 50 for OE and 100 for KD) were added to the RetroNectin-coated wells and centrifuged at 2,000*g* for 2 h at 32 °C. The supernatant was removed, and wells were washed once with PBS containing 2% BSA. Then, 0.5–1×10^4^ CD34^+^ cells (24 h pre-stimulated in HSC medium with UM171) were placed into lentivirus-preconditioned wells. At 16 h post-transduction, cells were washed from the RetroNectin with HSC medium and placed onto a fresh plate.

### Construction of lentiviral shGPRC5C

Short hairpin RNA vectors targeting GPRC5C were designed according to Pelossof et al.^53^. Lentiviral cloning was performed according to the protocol described in Fares et al.^3^.

Template from Pelossof et al.:

#### shGPRC5C.804

TGCTGTTGACAGTGAGCGCGTGGATCGTCATGTATACTTATAGTGAAGCCACAGATGTATAAGTATACATGACGATCCACATGCCTACTGCCTCGGA

#### shGPRC5C.528

TGCTGTTGACAGTGAGCGCAGAGGTCATCATCAATACAGATAGTGAAGCCACAGATGTATCTGTATTGATGATGACCTCTATGCCTACTGCCTCGGA

### Calcium flux assay

AML3 cells were infected with viral particles to overexpress GPRC5C or control. Cells were resuspended in IMDM with 1% FBS at 10^6^ cells ml^−1^. Per millilitre of cell suspension, 7.5 μl Indo-1 (Invitrogen) was added and incubated at 37 °C for 45 min. Following incubation, cells were washed twice with 1% FBS IMDM and then incubated for 30 min at 37 °C for de-esterification. The baseline of calcium was acquired on the LSR Fortessa II (BD Bioscience) for 30 s with the sample heater at 37 °C. The cells were stimulated with compounds and acquired for an additional 90 s. The ratio of indo-1-bound to indo-1-unbound fluorescence emission signals was calculated as a quantitative parameter for change in intracellular calcium levels. All values were normalized to the baseline of each sample.

### Isolation of human CD34^+^ cells

All experiments were performed according to the ethical protocols outlined in Bonn (AZ 264/18) and Mannheim (AZ 2020-639 N).

CD34^+^ cells were isolated using EasySep Human Cord Blood CD34 Positive Selection Kit II (StemCell Technologies). Cell pellets were collected and resuspended in buffer (PBS, 2 mM EDTA, 2% FBS and 1 μl ml^−1^ DNase) at 5 × 10^8^ cells ml^−1^. Immunomagnetic labelling and separation were performed according to the manufacturer’s instructions. Isolated cells were immediately stained with CD34/CD38 and sorted for CD34^+^CD38^−^ for downstream experiments.

### PRESTO-Tango assay

PRESTO-Tango assay was performed according to the protocol in Kroeze et al.^36^, with slight modifications. Briefly, HTLA cells were cultured in 10% FBS DMEM with 2 μg ml^−1^ puromycin and 100 μg ml^−1^ hygromycin B. Cells were transfected with 30 μg ml^−1^ of the GPRC5C-Tango plasmids using JetPrime. After 24 h of transfection, cells were plated at 60,000–80,000 cells per well into a 96-well plate. Then, 3.5× concentration of the drug was prepared in 20 mM HEPES and 1× HBSS, pH 7.4, and 40 μl of the drug population was added to each well. After 24 h of incubation, cells were washed with PBS and transferred into a 96-well plate (Greiner) with 40 μl of Bright-Glo solution (Promega). Following a 5-min incubation at RT, each well was measured for 5 s on a Centro LB 963 Microplate Luminometer (Berthold).

### In silico modelling

First, the three-dimensional coordinates of the 7TM regions of the protein were generated using homology modelling. In this approach, the sequences of the 7TM regions were retrieved from UniProtKB (ID: Q9NQ84-2), and suitable three-dimensional templates were identified using blastp. The three-dimensional structure of the 7TM was generated using Prime (Schrödinger Suite 2020-3). In the next step, we used the iterative threading assembly refinement (I-TASSER) server with an *ab initio* modelling protocol to generate the remaining residues of the ECD (amino acids 1–50) and the cytoplasmic domain (amino acids 301–441) (refs. ^54,55^). The best-ranked *ab initio* models were selected on the basis of the in-built ranking method and were added to the 7TM model using Modeller (Webb and Sali, 2016). The highest-ranked model was selected and energy-minimized in Prime using the OPLS3e force field (Schrödinger Suite 2020-3).

In the next step, the full-length model was subjected to 1 μs molecular dynamics simulations using the Desmond Molecular Dynamics System (Schrödinger Suite 2020-3). The system was built by adding highly aligned palmitoyl-oleoyl-phosphatidyl-choline bilayers to the full-length GPRC5C, which was placed in an orthorhombic water cell while applying the SPC solvent model and the OPLS3e force field. The overall system was neutralized by adding an appropriate number of Cl^−^ ions and was subjected to equilibration using the default Desmond relaxation protocol. The final state was subjected to 1 μs molecular dynamics in an *NpT* (constant temperature and constant pressure) ensemble at 300 K per 1 atm. The Nosé–Hoover chain and Martyna–Tobias–Klein with 1.0 ps and 2.0 ps relaxation time, respectively, and isotropic coupling were used. Coulomb interactions were evaluated using the smooth particle-mesh Ewald summation as a long-range method with 9 Å short-range cut-off. The representative structures were extracted from the overall timescale based on protein stability and subjected to further evaluation.

A representative structure was then selected from the overall trajectory based on the protein stability and subjected to extra-precision (XP) Glide docking (Schödinger Suite 2020-3) against HA using the default parameters. The best binding pose was selected on the basis of the highest XP Glide score. The images were created using PyMol (Schrödinger Suite 2020-3).

### scRNA amplification and library preparation

scRNA-seq was performed according to the mCEL-Seq2 protocol^20^. CD34^+^CD38^–^GPRC5C^pos/neg^ cells were sorted into 384-well plates containing 240 nl of unique primer mix and 1.2 μl mineral oil (Sigma-Aldrich). Sorted plates were centrifuged at 2,200*g* for 5 min at 4 °C and stored at −80 °C. Then, 160 nl reverse transcription reaction mix and 2.2 μl second-strand reaction mix (New England Biolabs) was added to each well to convert RNA into complementary DNA. cDNA from each plate was pooled and transferred to DNA LoBind microcentrifuge tubes (Eppendorf). AMPure/RNAClean XP beads (Beckman Coulter) (0.8× of total volume) were used for cDNA clean-up. In vitro transcription was done with the Transcription Kit (Thermo Fisher) at 37 °C for 13 h. To remove primers, samples were incubated with ExoSAP-IT (Thermo Fisher) at 94 °C for 3 min, and then a final RNA clean-up was performed with 0.8× RNAClean XP beads. Libraries were sequenced on a HiSeq 3000 (Illumina) and NovaSeq 6000 sequencing system (Illumina) (paired-end multiplexing run, high-output mode) at a depth of ~150,000–200,000 reads per cell.

LT-HSCs scRNA-seq was performed on the 10x Genomics platform using the Chromium Single Cell 3′ Reagent Kit V3 (10x Genomics) according to the manufacturer’s instructions. Briefly, 4,000 LT-HSCs cells were sorted and pooled from two CD34^+^ BM specimens (StemCell Technologies) into 0.4% BSA in PBS. Cells were loaded according to the manufacturer’s instructions, aiming for a targeted cell recovery of 2,000–3,000 cells. The quality of the obtained cDNA library upon adapter ligation and index PCR (13 cycles) was assessed by Bioanalyzer fragment analysis (High Sensitivity DNA Kit, Agilent). Libraries were sequenced on HiSeq 2500 and 3000 sequencing systems (Illumina) (paired-end multiplexing run, high output mode) at a depth of ~500,000 reads per cell.

### Bulk RNA-seq

The RNA of 1,000-3,000 freshly sorted cells was extracted using Arcturus PicoPure kit (Applied Biosystems) and DNase treatment was performed (Qiagen). Subsequently, cDNA libraries were prepared using SMART-Seq v4 Ultra Low Input RNA kit (Takara Bio) with 12 cycles of amplification. Further, NEBNext Ultra II FS DNA kit and NEBNext Multiplex Oligos (New England BioLabs) were used to generate uniquely dual-barcoded sequencing libraries from cDNA libraries. To this end, 5 ng of cDNA library was fragmented for 22.5 min, adaptors were ligated and libraries were amplified for eight cycles. RNA-seq libraries were sequenced at a depth of 55 million reads, 100 bp paired end on a NovaSeq platform (Illumina).

### Bulk RNA-seq analysis method: low-level processing

Raw fastq files were mapped against the hg38 or the mm10 reference genome by the mRNA-seq tool from the bioinformatics pipeline snakePipes^56^. ‘Alignment’ mode was used for mapping of sequenced reads using STAR (version STAR_2.7.4a)^57^, followed by the quantification of expression counts by featureCounts^58^. DeepTools QC (version 3.3.2) (ref. ^59^) was used for quality checking. Genes with an average expression that was higher than 100 counts among all samples were selected for further analysis. The differential expression was generated with DESeq2 (ref. ^60^); results with a false discovery rate <0.1 and a log_2_ fold change (FC) threshold ≥0.5 were considered significant.

### Bulk RNA-seq analysis method: downstream analysis

The relative expression of differentially expressed genes (DEGs) based on the variance-stabilized read counts as calculated by DESeq2 package was visualized with principal component analysis and heat maps. Volcano plots representing the results of differential expression were generated with EnhancedVolcano. GSEA was performed with the fgsea R package^61^ for evaluation of expression in pairwise comparisons of previously published HSCs activation/quiescence–dormancy gene signatures^11,21^, the hallmark gene set (h.all.v7.2.symbols.gmt^62^), the KEGG pathway database gene set (c2.cp.kegg.v7.2.symbols.gmt^63^) and the GO Biological Processes (c5.go.bp.v7.2.symbols.gmt,) obtained from the Molecular Signatures Database^64^, considering significant pathways at a false discovery rate <0.1. The GSEA enrichment profile of concrete signatures was plotted using gseaplot2 function.

### scRNA-seq analysis: quantification of transcript abundance

Paired-end reads from the mCEL-Seq2 and 10x human HSC data were aligned to the same reference genome (GRCh38) using the snakePipes scRNA-seq workflow (version 2.2.3) in Gruen mode and in STARsolo mode, respectively^56^. Gencode version 31 gtf was used for feature counting.

### Genotyping of patients in the 10x dataset and splitting cells by patient

To assign cells to the corresponding patient, genotyping procedure was used, as specified in Xu et al.^65^. Briefly, bam files produced by STARsolo were filtered for high-quality reads using samtools view parameters q 10 F 3844. Reads were assigned to genes with featureCounts using bam format as output. Gene-assigned bam files were de-duplicated per gene and per cell with umitools. Genetic variants were called on de-duplicated bams with freeBayes (with parameters -iXu -C 2 -q 1), and high-quality variants (QUAL >30) were filtered for with vcffilter. scSplit^65^ was run on the filtered vcf file, requesting two genotypes and setting a ceiling on the expected fraction of doublets at 20% (with parameters -n 2 -d 0.2).

Seurat object output by snakePipes (mode: STARsolo) was annotated with genotypes obtained from scSplit and split into three objects, containing counts for cells assigned to either of the two patients in the cell mixture, or cells predicted to be doublets. Unnormalized count matrices for the two patients were used in further analysis, while the doublet cells were discarded.

### scRNA sequencing data analysis

Overall, 1,827 cells passed the quality control threshold of >1,000 transcripts for the 10x Genomics human LT-HSC data. For normalization, the total transcript counts in each cell were normalized to 1, and were multiplied by the minimum total transcript count across all cells that passed the quality control threshold (>1,000 transcripts per cell). VarID^19^ was run with a vector of batch variables indicating the batches/patients, and the following parameters: mintotal = 1,000, minexpr = 5, minnumber = 5, large = TRUE, regNB = TRUE. For the mCEL-Seq2 datasets, we recovered 639 cells (mintotal = 1,000). VarID was run with the default parameters.

### Cell ordering and generation of SOMs

Cells were ordered in ascending order based on the coordinates of the first UMAP dimension, that is the cells’ *x*-axis coordinates from the UMAP. SOMs were generated using the FateID package based on the ordering inferred from the UMAP^20^. Only genes with more than two counts after size normalization in at least a single cell were included for the SOM analysis. In brief, smooth profiles were derived by applying local regression on normalized transcript counts after ordering cells. Next, a one-dimensional SOM with 200 nodes was computed on these profiles after *z*-transformation.

### Pathway enrichment analysis and GSEA

Symbol gene IDs were first converted to Entrez gene IDs using the clusterProfiler package^66^. Pathway enrichment analysis and GSEA^67,68^ were implemented using the ReactomePA package. Pathway enrichment analysis was performed on genes taken from the different modules in the SOMs. GSEA was performed using the DEGs between clusters 3, 2 and 8 and clusters 9, 4, 5 and 10 inferred by the diffexpnb function from the RaceID package.

### Human quiescent CB and dormant mouse HSC signatures

Quiescent CB genes were selected from the list of DEGs between quiescent and active HSCs from the bulk RNA-seq data generated in Belluschi et al.^11^. Genes with a log_2_FC >0.5 were selected for the quiescent signature, and their expression in the human 10x dataset was aggregated and plotted.

Dormant mouse HSC genes were selected from the list of DEGs between dormant and active mouse HSCs from the bulk RNA-seq data generated in Cabezas-Wallscheid et al*.*^21^. Human–mouse orthologous genes with a log_2_FC >1 were selected for the dHSC signature, and their expression in the human 10x dataset was aggregated and plotted.

### Statistics and reproducibility

Two-tailed *t*-tests were used to compare between groups, and the Mantel–Cox test was used to determine statistical significance of the survival curve. AT least two independent eperiments were performed for all experiments, unless otherwise stated. Data are shown as mean ± standard deviation (s.d.) or mean ± standard error of the mean (s.e.m.) as indicated. GraphPad Prism was used for statistical analysis.

### Reporting summary

Further information on research design is available in the Nature Research Reporting Summary linked to this article.

## Online content

Any methods, additional references, Nature Research reporting summaries, source data, extended data, supplementary information, acknowledgements, peer review information; details of author contributions and competing interests; and statements of data and code availability are available at 10.1038/s41556-022-00931-x.

## Supplementary information

Reporting Summary

Supplementary Table 1

## Data Availability

The raw data are deposited in ArrayExpress (http://www.ebi.ac.uk/arrayexpress) and are available under the accession numbers E-MTAB-9862, E-MTAB-9863,E-MTAB-9874, E-MTAB-9892, E-MTAB-9922 and E-MTAB-9967. Source data are provided with this paper. All other data supporting the findings of this study are available from the corresponding author on reasonable request.

## Source data

Source Data Fig. 1 Statistical source data.
Source Data Fig. 2 Statistical source data.
Source Data Fig. 3 Statistical source data.
Source Data Fig. 4 Statistical source data.
Source Data Fig. 5 Statistical source data.
Source Data Fig. 6 Statistical source data.
Source Data Extended Data Fig. 2 Statistical source data.
Source Data Extended Data Fig. 3 Statistical source data.
Source Data Extended Data Fig. 4 Statistical source data.
Source Data Extended Data Fig. 5 Statistical source data.
Source Data Extended Data Fig. 6 Statistical source data.
Source Data Extended Data Fig. 7 Statistical source data.
Source Data Extended Data Fig. 8 Statistical source data.
