# Supplementary material for: Hyaluronic acid–GPRC5C signalling promotes dormancy in haematopoietic stem cells
Source: Nat Cell Biol. 2022 Jun 20;24(7):1038–48. doi: 10.1038/s41556-022-00931-x (PMC9276531; doi:10.1038/s41556-022-00931-x)
Supplement: Supplementary file 1 — Reporting Summary [file 41556_2022_931_MOESM1_ESM.pdf]

nature research

Corresponding author(s): Nina Cabezas-Wallscheid

Last updated by author(s): 14/04/2022

Reporting Summary

Nature Research wishes to improve the reproducibility of the work that we publish. This form provides structure for consistency and transparency in reporting. For further information on Nature Research policies, see our Editorial Policies and the Editorial Policy Checklist.

Statistics

For all statistical analyses, confirm that the following items are present in the figure legend, table legend, main text, or Methods section.

n/a	Confirmed	
☐	☒	The exact sample size (*n*) for each experimental group/condition, given as a discrete number and unit of measurement
☒	☐	A statement on whether measurements were taken from distinct samples or whether the same sample was measured repeatedly
☐	☒	The statistical test(s) used AND whether they are one- or two-sided<br>*Only common tests should be described solely by name; describe more complex techniques in the Methods section.*
☐	☒	A description of all covariates tested
☐	☒	A description of any assumptions or corrections, such as tests of normality and adjustment for multiple comparisons
☐	☒	A full description of the statistical parameters including central tendency (e.g. means) or other basic estimates (e.g. regression coefficient) AND variation (e.g. standard deviation) or associated estimates of uncertainty (e.g. confidence intervals)
☐	☒	For null hypothesis testing, the test statistic (e.g. F, t, r) with confidence intervals, effect sizes, degrees of freedom and P value noted<br>*Give P values as exact values whenever suitable.*
☒	☐	For Bayesian analysis, information on the choice of priors and Markov chain Monte Carlo settings
☐	☒	For hierarchical and complex designs, identification of the appropriate level for tests and full reporting of outcomes
☒	☐	Estimates of effect sizes (e.g. Cohen's *d*, Pearson's *r*), indicating how they were calculated

Our web collection on statistics for biologists contains articles on many of the points above.

Software and code

Policy information about availability of computer code

Data collection

BD FACSDiva v8.0.3 (Flow cytometers and sorters, BD Bioscience), ZEN blue v2.5 (Zeiss international), Berthold Microplate Luminometer (Berthold), confocal microscope (Nikon C2).

Data analysis

Bulk RNA-seq analysis method: low level processing
Raw fastq files were mapped against the hg38 or the mm10 reference genome by the mRNA-seq tool from the bioinformatics pipeline snakePipes. “Alignment” mode was used in this tool for the mapping of sequenced reads using STAR (STAR_2.7.4a), followed by the quantification of expression counts by featureCounts. Deeptools QC (3.3.2) was used for quality checking. Genes with an average expression higher than 100 counts along all samples were selected for further analysis. The differential expression was generated with DESeq; considering significant the results with a FDR < 0.1 and a log2FC threshold ≥ 0.5.
Bulk RNA-seq analysis method: Downstream analysis
The relative expression of DEGs based on the variance stabilized read counts calculated by DESeq2 package was visualized in PCA and heatmaps. Volcano plots representing the results of differential expression were generated with EnhancedVolcano. Gene set enrichment analysis (GSEA) was performed with fgsea R package for evaluation of expression in the pairwise comparisons of previously published gene signatures of HSCs Activation/ Quiescence-Dormancy, the hallmark gene set (h.all.v7.2.symbols.gmt), the KEGG pathway database gene set (c2.cp.kegg.v7.2.symbols.gmt) and the GO Biological Processes (c5.go.bp.v7.2.symbols.gmt,) obtained from the Molecular Signatures Database, considering significant pathways at an FDR < 0.1. The GSEA enrichment profile of concrete signatures was plotted using gseaplot2 function.

Single-cell RNA-seq analysis
Quantification of transcript abundance
Paired-end reads from the mCEL-Seq2 and 10x human HSC data were aligned to the same reference genome (GRCh38) using snakePipes scRNAseq workflow in mode Gruen and in mode STARsolo, respectively. Gencode version 31 gtf was used for feature counting.

Genotyping of patients in the 10x dataset and splitting cells by patient:

To assign cells to the corresponding patient, genotyping procedure as specified on was used. Briefly, bam files produced by STARsolo were filtered for high quality reads using samtools view parameters q 10 F 3844. Reads were assigned to genes with featureCounts using bam format as output. Gene-assigned bam files were deduplicated per gene and per cell with umi tools. Genetic variants were called on deduplicated bams with freeBayes (with parameters -iXu -C 2 -q 1), and high quality variants (QUAL > 30) were filtered for with vcffilter .scSplit was run on the filtered vcf file requesting two genotypes and setting a ceiling on the expected fraction of doublets at 20% (parameters -n 2 -d 0.2).
Seurat object output by snakePipes mode STARsolo was annotated with genotypes obtained from scSplit and split into three objects, containing counts for cells assigned to either of the two patients in the cell mixture, or cells predicted to be doublets. Unnormalized count matrices for the two patients were used in further analysis, while the doublet cells were discarded.
Single-cell RNA sequencing data analysis
Overall, 1,827 cells passed the quality control threshold of >1,000 transcripts for the 10x Genomics human LT-HSC data. For normalization, the total transcript counts in each cell were normalized to 1 and multiplied by the minimum total transcript count across all cells that passed the quality control threshold (>1,000 transcripts per cell). VarID19 was run with a vector of batch variables indicating the batches/patients, and the following parameters: mintotal = 1000, minexpr = 5, minnumber = 5, large=TRUE, regNB=TRUE. For the mCEL-Seq2 datasets, we recovered 639 cells from patient 1048 (mintotal=1000). VarID was run with the default parameters.
Cell ordering and generation of self-organizing maps
Cells were ordered in ascending order based on the coordinates of the first UMAP dimension, i.e., the cells' x-axis coordinates from the UMAP. SOMs were generated using the FateID package on the basis of the ordering inferred from the UMAP. Only genes with >2 counts after size normalization in at least a single cell were included for the SOM analysis. In brief, smooth profiles were derived by applying local regression on normalized transcript counts after ordering cells. Next, a one-dimensional SOM with 200 nodes was computed on these profiles after z-transformation. Neighboring nodes were merged if the Pearson's correlation coefficient of the average profiles of these nodes exceeded 0.85. The remaining aggregated nodes represent the gene modules shown in the SOM figures.

Pathway enrichment analysis and gene set enrichment analysis.
Symbol gene IDs were first converted to Entrez gene IDs using the clusterProfiler package. Pathway enrichment analysis and GSEA were implemented using the ReactomePA package. Pathway enrichment analysis was done on genes taken from the different modules in the SOMs. GSEA was done using the differentially expressed genes between clusters 3,2,8 and clusters 9,4,5,10 inferred by the diffexpnb function from the RaceID package.

Human Quiescent CB and dormant mouse HSC signatures
Quiescent CB genes were selected from the list of differentially expressed genes between quiescent and active. HSCs from the bulk RNA-seq data generated in Belluschi et al.. Genes with a log2 FoldChange > 0.5 were selected for the quiescent signature, and their expression in the human 10x dataset was aggregated and plotted.
Dormant mouse HSC genes were selected from the list of differentially expressed genes between dormant and active mouse HSCs from the bulk RNA-seq data generated in Cabezas-Wallscheid et al.,. Human-mouse orthologous genes with a log2 FoldChange > 1 were selected for the dormant HSC signature, and their expression in the human 10x dataset was aggregated and plotted.

We used the following tools for analysis of bulk RNA-seq:
-snakePipes mRNA-seq workflow in mode Alignment and STAR for preprocessing
-R package DESeq2 for differential expression; considering significant the results with a FDR < 0.1 and a log2FC threshold ≥ 0.5.
-R package EnhancedVolcano for volcano plots representation
-R packages fgsea and enrichplot for GSEA

We used the following tools for analysis of single-cell RNA-seq:
-snakePipes scRNAseq workflow in mode Gruen and STARsolo for preprocessing of mCEL-Seq2 dataset
-STARsolo, samtools, featureCounts and umi tools for preprocessing of 10x dataset
-freeBayes for genetic variants calling
-VarID for batch removal
-FateID package for SOMs generation
-R package clusterProfiler for symbol gene IDs to Entrez gene IDs conversion
-R package ReactomePA for pathway enrichment and GSEA

For manuscripts utilizing custom algorithms or software that are central to the research but not yet described in published literature, software must be made available to editors and reviewers. We strongly encourage code deposition in a community repository (e.g. GitHub). See the Nature Research guidelines for submitting code & software for further information.

Data

Policy information about availability of data

All manuscripts must include a data availability statement. This statement should provide the following information, where applicable:
- Accession codes, unique identifiers, or web links for publicly available datasets
- A list of figures that have associated raw data
- A description of any restrictions on data availability

RNA-seq data has been deposited on ArrayExpress (http://www.ebi.ac.uk/arrayexpress) under the following accession numbers: E-MTAB-9862, E-MTAB-9863, E-MTAB-9874, E-MTAB-9892, E-MTAB-9922, E-MTAB-9967

Field-specific reporting

Please select the one below that is the best fit for your research. If you are not sure, read the appropriate sections before making your selection.

☒ Life sciences ☐ Behavioural & social sciences ☐ Ecological, evolutionary & environmental sciences

Life sciences study design

All studies must disclose on these points even when the disclosure is negative.

Sample size	Sample size was determined based on extensive experience with similar experiments in our laboratory (Cabezas-Wallscheid et al., 2014 and 2017; Sommerkamp et al., 2020 and 2021; Renders et al., 2021)
Data exclusions	Sample exclusion was done only as a result of premature mouse death or if clear errors in pre-processing occured.
Replication	All attempts of replication were successful. Key experiments were performed at least twice, exact number of independent experiments with various biological replicates can be found in the figure legends.
Randomization	All samples/ mice were analysed and allocated randomly.
Blinding	No blinding experiments were needed, since values were quantitative comparisons as determined by software and measurement.

Reporting for specific materials, systems and methods

We require information from authors about some types of materials, experimental systems and methods used in many studies. Here, indicate whether each material, system or method listed is relevant to your study. If you are not sure if a list item applies to your research, read the appropriate section before selecting a response.

Materials & experimental systems

n/a	Involved in the study	
☐	☒	Antibodies
☐	☒	Eukaryotic cell lines
☒	☐	Palaeontology and archaeology
☐	☒	Animals and other organisms
☒	☐	Human research participants
☒	☐	Clinical data
☒	☐	Dual use research of concern

Methods

n/a	Involved in the study	
☒	☐	ChIP-seq
☐	☒	Flow cytometry
☒	☐	MRI-based neuroimaging

Antibodies

Antibodies used

Human Antibodies
APC-Cy7 CD34 BioLegend 343514 1/100,
PE CD90 BioLegend 328110 1/50,
BV605 CD90 BD Biosciences 562685 1/50,
PE-Cy7 CD38 BioLegend 303516 1/100,
PE CD201 BioLegend 351904 1/50,
BV421 CD45RA BD Biosciences 562885 1/100,
FITC GPRC5C R&D Systems FAB6594G-100UG 1/50 Clone:577315,
AF700 GPRC5C R&D Systems FAB6594N-100UG 1/50 Clone: 577315,
FITC CD45RA BD Biosciences 555488 1/100,
PE-Cy5 CD49f BD Biosciences 551129 1/200,
APC CD201 BioLegend 351906 1/50,
PE Ki67 BioLegend 350504 1/200,
PE-Cy5 Ki67 BioLegend 350519 1/200,
AF488 Ki67 BioLegend 350507 1.200,
AF647 CDK6 Abcam ab198946 1/500,
PE CD33 BD Biosciences 555450 1/200,
PE-Cy7 CD19 BD Biosciences 557835 1/200,
BV421 CD34 BD Biosciences 744904 1/100,
BV421 CD66b BD Biosciences G10F5 1/100 ,
BV650 CD15 BioLegend 323033 1/200,
BV711 CD14 BioLegend 367139 1/1000,
FITC CD56 BioLegend 362545 1/200,
PE-Cy5 CD45 BioLegend 304009 1/300,
PE CD71 BioLegend 334105 1/1000,
PE-Cy7 GlyA BioLegend 334619 1/300,
APC CD41a BioLegend 303709 1/200,
APC-Cy7 CD11b BioLegend 101225 1/300,
Hyaluronan Binding protein Amsbio AMS.HKD-BC40 1/50.
Hyaluronic Acid LS Bio C295845 1/50
Flag Tag clone M2 Sigma F3165

Mouse Antibodies
BV650 CD8a BioLegend 100742 1/1000,
BV650 CD11b BioLegend 101259 1/1000,
BV650 Ly-6G/Ly-6C (Gr-1) BioLegend 108442 1/1000,
BV650 TER-119/Erythroid Cells BioLegend 116235 1/1000,
BV650 CD45R/B220 BioLegend 103241 1/1000,
BV650 CD4 BD Biosciences 563232 1/1000,
PE/Cy7 anti-mouse CD8a Biolegend 100722 1/1000,
PE/Cy7 anti-mouse/human CD11b Biolegend 101216 1/1000,
PE/Cy7 anti-mouse Ly-6G/Ly6C (Gr-1) Biolegend 108416 1/1000,
PE/Cy7 anti-mouse TER-119/Erythroid Cell Biolegend 116221 1/1000,
PE/Cy7 anti-mouse/human CD45R/B220 Biolegend 103222 1/1000,
PE/Cy7 anti-mouse CD4 Biolegend 100422 1/1000,
Brilliant Violet 711 anti-mouse CD117 (c-kit) Biolegend 105835 1/1000,
Brilliant Violet 421 anti-mouse CD117 (c-kit) Biolegend 105828 1/1000,
PE anti-mouse CD117 (c-kit) Biolegend 105808 1/100,
APC/Cy7 anti-mouse Ly-6A/E (Sca-1) Biolegend 108126 1/500,
PE/Cy5 anti-mouse 150 (SLAM) Biolegend 115912 1/500,
Pacific Blue anti-mouse CD48 Biolegend 103418 1/500,
APC-eFlour780 CD45 Invitrogen 47-0451-82 1/200,
PE Streptavidin Biolegend 405203 1/2000,
CD34-AF700 Biolegend 560518 1/50,
CD34-FITC Biolegend 553733 1/50,
CD45.2-Pacific Blue Biolegend 109820 1/500,
CD45.1-PE/Cy7 Biolegend 110730 1/500,
CD4-PE/Cy5 Biolegend 100410 1/2000,
CD8a-PE/Cy5 Biolegend 100710 1/1000,
CD11b-APC/Cy7 Biolegend 101226 1/1000,
Gr1-APC Biolegend 108412 1/1000,
B220-AF700 Biolegend 103434 1/500

For flow cytometry antibodies, Lot numbers cannot reasonably be provided, as multiple different lots have been used over the course of this study. Depending on the individual experimental setting flow cytometry antibodies from the same clone but coupled to different fluorochromes were used.

Validation

All flow cytometry antibodies were already established and commonly used in our laboratory and have been published multiple times by us and other groups. CDK6 was validated on more vs. less quiescent cells as described in Cabezas-Wallscheid et al., 2017, Cell. GPRC5C (R&D) was extensively validated as shown in Extended Data Figure 2.

Eukaryotic cell lines

Policy information about cell lines

Cell line source(s)

AML3 (Max Planck), HEK293T (Takarabio), AML5 (DSMZ)

Authentication

Common and well-established cell lines. None of the cell lines were authenticated.

Mycoplasma contamination

Not detected

Commonly misidentified lines
(See ICLAC register)

No commonly misidentified lines were used

Animals and other organisms

Policy information about studies involving animals; ARRIVE guidelines recommended for reporting animal research

Laboratory animals

NOD,B6.SCID Il2rγ−/−KitW41/W41 Jackson 26497
C57BL/6J (CD45.2) MPI-IE
B6Ly5.1 (CD45.1) MPI-IE
B6Ly5.1(CD45.1/2) MPI-IE
Gprc5c LacZ Knockin Mice (Gpr5c-KO) Sano et al.
B6.129(Cg)-Cd44tm1Hbg/J Jackson 005085

Male and female animals were used. Mice were between 8-weeks to 12-weeks of age.

Wild animals

No wild animals were used in this study

Field-collected samples

No field collected samples were used in this study

Ethics oversight

All mice were bred in-house in the animal facility at the MPI-IE in individually ventilated cages (IVCs). According to German guidelines, mice were euthanized by cervical dislocation. Animal procedures were performed according to the protocols

approved by the German authorities, Regierungspräsidium Freiburg (the sacrificing of animals for scientific purposes according to §4 (3) of the German Animal Protection Act; animal protocol numbers: G-18/142, G-19/74, G20/124

Note that full information on the approval of the study protocol must also be provided in the manuscript.

Flow Cytometry

Plots

Confirm that:

☒ The axis labels state the marker and fluorochrome used (e.g. CD4-FITC).

☒ The axis scales are clearly visible. Include numbers along axes only for bottom left plot of group (a 'group' is an analysis of identical markers).

☒ All plots are contour plots with outliers or pseudocolor plots.

☒ A numerical value for number of cells or percentage (with statistics) is provided.

Methodology

Sample preparation

Cell cycle analysis (Ki-67 or CDK6)
Stained cells were fixed with BD Cytofix/Cytoperm Buffer (BD Biosciences) for 10 minutes at 4 °C. Next, cells were stained with intracellular Ki-67 at 1:100 (BD Biosciences) or CDK6 at 1:500 (Abcam) in PermWash solution (BD Biosciences) for 45–90 minutes at 4 °C. Prior to cell cycle analysis on the BD LSRFortessa Cell Analyzer (BD Biosciences), cells were stained in the dark with Hoechst 33342/DAPI (Invitrogen) for 30 minutes at RT.

CellROX/MitoTracker staining
Cells were incubated at 37 °C with CellROX DeepRed at 1/500 (Invitrogen) or MitoTracker Red at 50 nM (Invitrogen) in their corresponding media for 30 minutes. Cells were subsequently washed 3 times in PBS and stained for FACS analysis on the BD LSRFortessa Cell Analyzer (BD Biosciences).

O-propargyl-puromycin
Stained cells were fixed for 15 minutes at RT with BD Cytofix/Cytoperm Buffer (BD Biosciences) and permeabilized with 0.1% Triton for 15 minutes at RT. The copper-catalysed azide-alkyne cycloaddition (CuAAC) was performed using an Alexa594-azide (Life Technologies, 5 mM final concentration) and the Click-iT Cell Reaction Buffer Kit (Life Technologies) according to the manufacturer's instructions.

HA-fluorescein binding assay
HEK293T cells were transfected with 30 µg/ml of the GPRC5C-Tango plasmids using JetPrime transfection reagent. After 24 to 36 hours' transfection, 60,000 to 80,000 cells were treated with 8 µg HA conjugated to fluorescein with or without, 100 µM NAG for 60 minutes at RT with shaking. Cells were washed and fluorescein was quantified using the LSR II flow cytometer (BD Bioscience).

Calcium flux assay
AML3 cells were infected with viral particles to overexpress GPRC5C or control. Cells were resuspended in IMDM with 1% FBS at 106 cells/ml. 7.5 µl Indo-1 (Invitrogen) was added per millilitre of cell suspension and incubated at 37 °C for 45 minutes. Following incubation, cells were washed twice with 1% FBS IMDM and then incubated for 30 minutes at 37 °C for de-esterification. The baseline of calcium was acquired on the LSR Fortessa II (BD Bioscience) for 30 seconds with the sample heater at 37 °C. The cells were stimulated with compounds and acquired for an additional 90 seconds. The ratio of indo-1-boundsto indo-1-unbound fluorescence emission signals was calculated as a quantitative parameter for change in intracellular calcium levels. All values were normalized to the baseline of each sample.

Instrument

For cell sorting: FACS Aria Fusion (Becton Dickinson)
For analysis: LSRII, LSR Fortessa(Becton Dickinson)

Software

Analysis was performed with FlowJo, statistical analysis with Graphpad Prism.

Cell population abundance

Population abundance is reported on Figures.

Gating strategy

Phenotype Gating:
FSC-A v. SSC-A (cell gate excluding debris) --> FSC-W v. FSC-H (doublet exclusion)--> SSC-W v. SSC-H (doublet exclusion) --> more detailed gating strategy for each individual experiment is indicated in Supplemental Information

Cell Cycle Gating:
FSC-A v. SSC-A (cell gate excluding debris) --> DAPI-A v. DAPI-H (doublet exclusion) --> gating in interested population --> Ki-67 and DAPI-H.

☒ Tick this box to confirm that a figure exemplifying the gating strategy is provided in the Supplementary Information.
